# Supplementary material for: Lipid gating of BK channels and activation mechanism by negatively charged lipids
Source: Commun Chem. 2026 Oct 6;9:315. doi: 10.1038/s42004-026-02245-7 (PMC13642915; doi:10.1038/s42004-026-02245-7)
Supplement: Supplementary file 1 [file 42004_2026_2245_MOESM1_ESM.pdf]

nature portfolio

Peer Review File

Lipid gating of BK channels and activation mechanism by negatively charged lipids

Corresponding Author: Dr Wojciech Kopec

This file contains all reviewer reports in order by version, followed by all author rebuttals in order by version.

Version 0:

Reviewer comments:

Reviewer #1

(Remarks to the Author)
This is an interesting computational study of the role of lipids in gating of the BK channel. The authors have performed extensive atomistic and coarse-grained simulations of the channel in various state (Ca binding, voltage) and lipid environment (w/ and w/o POPS) to investigate the conformational dynamics of the channel and how lipid fenestration may play a central role in driving the hydrophobic gating of the channel. Even though lipid fenestration has been observed and discussed in several studies perviously, this is one of the first studies that focus on the role of lipid fenestration in BK gating. The central conclusion is that lipid fenestration is a critical determinant of BK conductivity. The authors further proposed that negatively charged lipids such as POPS could reduce lipid fenestration and promote channel activation. I think these are valuable insights that could add to the mechanistic understanding of BK channels. However, I also have several important concerns for the authors to consider.

1) It is curious and at the same time quite troubling that the channel display significant instabilities in the simulations reported in this work. This is particularly surprising and concerning in the simulation of the open, Ca2+-bound state. It is well established experimentally that the calcium bound state of the channel should remain stably open. The strange blockage of channel by lipid in Ca2+ bound and 300 mV should be considered a clear artifact of the simulation, instead of reliable refection of how the channel behave. The observation that the full lipid could enter and occupy the pore is extremely suspicious, and to me, is a sign of simulation artifacts. There is no evidence from any experiment that would support anything beyond lipid tail fenestration.

2) The above examples are quite representative of the most important reservation that I have for this work. The authors seem to adopt a dangerous approach of taking everything observed in simulations at face value. Despite important advances in the reliability of atomistic simulation, this approach can easily lead to misleading and oftentimes completely wrong conclusions. Coupled with the seemingly blind trust of simulation, there are dangerous signs of confirmation bias when discussing experimental observations. I think it is crucial to take a much more critical approach in analysis and interpretation of the simulations.

3) Previous simulations by Chen and Cui labs suggest that pore dehydration does not depend on lipid fenestration, even though free simulations do observe dynamic lipid fenestration coupled with pore dehydration. This seems to argue that, despite the correlation between pore hydration and lipid fenestration, dehydration is the driving event and lipid fenestration is not. If the authors want to argue the opposite, control simulations with lipids excluded from fenestration much be performed to test if fenestration is indeed required. Importantly, the channel must be unrestrained to allow conformational response to dehydration of the pore. It is interesting to note that the Martini simulations seem to suggest that lipid fenestration can only occur if the pore is forced to dehydrate, even though that these coarse-grained simulations need to be taken with a large grain of salt.

4) I have strong reservations on the use of Martini simulations to examine detailed interactions and fenestration of lipids with ion channels. The lipid tails (and waters) are too “fat” due to the spherical representation of 4 CH2 or H2O with a single bead. Such coarse-graining severely distort the geometry fit of the tail (and water) in fenestration window and the pore. The fact that Martini simulations fail to observe pore dehydration is a clear indication of the limitation of Martini simulations in examining such detailed questions. The difference between pore occupancy is insignificant (e.g., Fig 3B) and there is great danger in interpreting these small differences.

Reviewer #2

(Remarks to the Author)
Paper Review

Summary
The manuscript broadly addresses BK channel opening and closing mechanisms facilitated by lipids using MD simulations to observe atom and near-atom level dynamic motions of the channel.
The authors investigate the concept of “physiological” BK closed state, which appears to be absent from experimental structures, and focus on the role of lipid tails during the pore dehydration event, previously observed in MD simulations of an isolated pore domain. The authors show that lipids are able to block the pore due to lipid tails entering fenestrations in the pore, and causing pore dehydration. The authors apply an electric field across the membrane in order to be able to classify the Ca-bound, hydrated pore structure as ‘open’, and the Ca-free, lipid-blocked as closed, and also classify a third partially blocked Ca-free state with intermediate conductance. The authors offer an explanation for the experimentally observed phenomenon that charged lipids play a role in regulating channel opening, by showing that PS is less likely to block the pore, and further decreases the number of potassium ions in the pore. The paper therefore addresses two separate, but related concepts – dehydration and closure of the channel correlated with lipid tail entry into the pore, and channel activation mediated by negatively charged lipid headgroups.

The introduction gives a comprehensive summary of the state-of-the-art. The authors are to be commended on the range of simulation techniques used. The findings are certainly exciting in terms of furthering our understanding of the complexity of lipid regulations of membrane protein function. The results are compelling and justify the conclusions drawn. At times the presentation of the results could be a little clearer, with specifics detailed below.

There are a number of points:
Intro:
It would be nice to have an idea of the protein primary expression tissue and the role it has in the organism.

Methods:
What is the reason for running the simulations at 323 K (50 °C)?

Results:
It would be helpful to have a short summary of the simulations performed at the start of each results section.

The use of the word ‘replica’ is a little confusing, as it might mislead the reader to think that the simulations were run in the replica-exchange regime. The authors might consider using the word ‘repeat’ instead.

Section on all-atom simulations of calcium-bound and calcium-free states: According to Table S1, calcium-bound and calcium-free states with applied voltage were run with 6 repeats. Why can we only see three repeats for each setup in Fig 1C, D.

Might the authors consider specifically referring to which repeat is being discussed, eg: as int the ms:“spontaneous dehydration events under applied voltage (2 out of 6 replicas), and a decrease in pore hydration in one equilibrium replica out of 5 (Fig. 1B, SI Fig. 1B)”. It is not clear which of the repeats is the single sponateous dehydration event (end of Rep2?)

And also here: from the ms: “the Ca2+-free channel showed a dehydrated pore in all simulations except 1) one equilibrium replica out of five”. In Fig 1A it looks like rep 1 and rep5 are dehydrated – could this be clarified?

“in the simulations of the Ca2+-free state at +300 mV where the pore remained hydrated and not blocked by lipids, the channel showed steady outward K+ permeation”

Could the conductance of Ca-free, fully hydrated at +300mV be added as a third bar in Fig 1D?

In the CHARMM36m simulations of the Ca-bound, did the eight calcium ions remain bound over the course of the simulation?

Discussion:
Could the authors comment on how the inclusion of cholesterol might have altered the results, since this is a major component of the plasma membrane. Was there a rationale to not including cholesterol?

Could the authors comment on how the choice of atomistic force field might have affected the results (eg CHARMM36m vs AMBER)? A paper from 2020 (https://doi.org/10.1021/acs.jctc.0c00331) reports notable differences in pore stability in Charmm36 vs. Amber simulations of potassium channels.

In the Martini3 simulations, the dehydrated pore has, on average 25 lipid beads in the pore, which corresponds roughly to 75-100 non-H atoms (although difficult to be sure of this conversion without knowing the Martini bead types). This is higher than the number of lipid atoms in the pore in the Charmm36m simulations (Fig1B, Fig 2A from 800-1000 ns). Could the authors justify this?

Figures:
Overall, the figures could be made more helpful in terms of understanding the results presented by the authors. In their current state, the figures are disjointed, require repeated and laborious cross-referencing with the supplementary figures, and are not self-sufficient, but instead rely heavily on the explanations for the main text. For example:

The overview of the structure presented in Figure 1A is useful and an excellent inclusion, and it would be useful to zoom in on the pore area, to show this in clearer detail.

Fig 1B: it is not clear if these graphs show data from all simulations, or for a single repeat. This could be made more explicit.

It would be helpful if eg helix S6 were labelled in Fig 1C

Fig 1E, S2a,C: what is the reason for using radians? Degrees would be perhaps more conventional.

Fig2C, D: the structure representation could perhaps be included to make clearer, eg render the helices with a less transparent material. It would also be helpful to note in the figure legend that F304 is coloured red.

Fig. 3D only makes sense when compared to uncharged lipid densities (Fig. S7A), so both should be presented in the same figure.

It should be made more explicit in the figure legend why 1/3 of POPE is being shown in Fig. 4B.

Reviewer #3

(Remarks to the Author)
Based on detailed molecular simulations, the present manuscript strongly argues in favor of lipid gating of BK channels. Previous work, including studies by one of the authors of the present manuscript, has been in favor of BK channel closure mediated by a hydrophobic gating mechanism. This type of gating was mediated by a decrease in the internal cavity diameter and an increase in its hydrophobicity, resulting from the rotation of the S6 transmembrane segment and the exposure of amino acid residues F304 and I308 to the cavity. The authors now contest that even the narrowest internal BK cavity of a closed BK channel reported in a recent paper (Contreras et al. 2025. Nature Communications) is not narrow enough to hinder K+ conduction. Thus, to promote pore dehydration and close the channel, lipid must enter through the channel fenestrations present only in closed BK channels. Given the unrestricted conditions for lipid movement through the fenestrations during the simulations, the results presented are convincing and show that a lipid block can be partial or, if the lipid occupies the internal cavity, complete. This observation leads to interesting consequences regarding the dwell time of the closed states. Of note is the finding that pore dehydration was always accompanied by the entrance of lipids to the pore. The authors further determine the ability of the BK channel to conduct ions by performing simulations in the presence of a 300 mV applied voltage. The effect of membrane lipid composition was also studied, concluding that lipid pore occupancy is reduced when the membrane composition is a mixture of neutral and negatively-charged lipids (POPE:POPS).
Comments
1. The paper by Coronel et al. (2024) not only suggested the necessity of lipid entry for pore dehydration, but their findings also led them to the conclusion that gating was controlled by lipid entrance through the lateral fenestrations. Even the title of that paper is not very different from that of the manuscript by Minorenko et al. The authors cannot downplay the importance of this previous work. They should establish in the discussion the novelty of their study and the contribution to a further understanding of BK lipid gating.
2. The sentence in the Introduction, “In a recent CryoEM study, mutations of voltage-sensing arginines that promote deactivation made it possible to obtain structures with the pore as narrow as ~8 Å in diameter, which nevertheless can still fit a hydrated K+ ion” is misleading. The evidence suggest that a pore ~8 Å in diameter can be dehydrated if it is hydrophobic enough. Given the structural results of Contreras et al. (2025), which show that the internal vestibule becomes quite hydrophobic upon channel closure, the importance of pore diameter and hydrophobicity should be discussed in more detail.
3. One of the novelties of the present study is the simulations in the presence of an applied voltage to determine ion conduction. However, Paulo et al. (Nature Communications 2023) discussed the effect of applied voltages on nanopore hydration and tested their predictions by engineering a biological nanopore. It would increase the importance of the author´s findings if they confronted their results with those of Paulo et al.
4. In the Introduction and Discussion, the paper by the Lingle´s group is cited, pointing out that large organic blockers cannot block fully closed channels . The authors should be aware that Contreras et al. (2025) found, using the structure of a "fully closed" BK channel (R3Q), that hindrance to blockade is due to a decreased pore diameter. The closure at I308 is sufficient to occlude entry of bbTBA into the R3Q apo channel.
5. The authors' simulations, conducted in the presence of a 300 mV applied voltage, demonstrate a full lipid block. However, at 300 mV and in the absence of Ca2+, the probability of opening is essentially 1. How do they explain this discrepancy between the simulation and the electrophysiological result? According to the simulations, one would expect a much lower open probability.
6. The finding of a lower single-channel conductance in the Ca2+-free than in the Ca2+-bound condition is also at odds with the experimental result that shows that the BK single-channel conductance in the absence of Ca2+ is not different from that in the presence of the divalent cation (see Taladucker and Aldrich, Biophys J. 2000).

A note for the authors regarding the effect of charged lipids.
The results obtained in the presence of negatively charged lipids are exciting. They may solve an old conundrum regarding the effect of charged lipids on the probability of opening (Po) and the channel conductance. Note that Moczydlowski et al. (1985, J.Membr. Biol.) determined the effect of the charge of the lipid (PS)on the BK single-channel conductance and Po. They concluded that the surface potential generated by the charged bilayer was responsible for the effects found on channel conductance and Po. However, Modzydlowski et al. (2003, J. Gen. Physiol.) found PS lipid bilayers were able to modulate channel conductance but not external Ba2+ and TEA blockade. Thus, the surface potential of the bilayer induced by the negative charge of the bilayer cannot explain these results.
My point here is that the results presented in this paper provide a parsimonious explanation for these contradictory results. If PS can reach the internal cavity, it should affect conductance and blockade by internal Ba2+.

Minor
1. The descriptions of the figures are confusing. For example, it is unclear why the authors started describing Fig. 1B instead of Fig.. 1A. Although it is true that in the calcium-free condition the pore is dehydrated in replicas 1 and 5, it contains a considerable number of water molecules in 3 and 4. So the question is how the authors define a dehydrated non-conductive pore. Considering that Fig. 1C and SI Fig. 3 show that a channel with 40 or less water atoms is conductive. Please clarify this point.
2. Please indicate in the text to which replicas you are referring, as in many cases, it is simply stating “the replica shows” without a number.
3. The ordinate given in number of atoms is confusing. For example, if, as the legend says, "Times traces of the number of lipid (non-hydrogen) atoms, the reader would like to know how many lipids the number of atoms represents.
4. Fig. 2A and SF Fig. 3 top show the same replicas. This is confusing.
5. For consistency, the authors should use either the number of atoms or the number of water molecules in the internal cavity (cf. SI Fig. 1 and Fig. SI Fig. 3).

Reviewer #4

(Remarks to the Author)
I co-reviewed this manuscript with one of the reviewers who provided the listed reports. This is part of the Communications Chemistry initiative to facilitate training in peer review and to provide appropriate recognition for Early Career Researchers who co-review manuscripts.

Version 1:

Reviewer comments:

Reviewer #1

(Remarks to the Author)
The authors' strong efforts to address my previous comments are greatly appreciated. Many of the changes made have improved the manuscript. However, my following major reservations remain.

1) The authors stated that full lipid molecules have been observed in the pore K2P channels (Ma et al, 2026). I actually could not find such data/conclusion in my reading of the same paper. Even if what the authors stated is true, K2P is quite different from BK. None of ~10 BK structures from several independent labs has ever observed densities that could suggest a full lipid in the pore. There is also minimal lipid density (or fenestration window) in the open state structures. The experimental evidence is missing to support the possibility of full lipid occupancy in the pore; instead, all evidence supports that only lipid tails can enter the pore transiently.

2) It is interesting that the authors discovered that Ca2+ was lost in some of the simulations of the open state w/ Ca2+ bound and 300 mV, which may explain instabilities in these trajectories. However, Ca2+ binding to CTD should be very tight and the observation that Ca2+ was lost itself is a huge red flag (and not reported by any of the published atomistic simulations). My honest take is that many things could go wrong and lead to "surprising" and often confusing observations in atomistic simulations. Most of the time, these are due to technical reasons (such as inadequate equilibration, artifacts in the initial structure and numerous other possibilities). I think it is dangerous to trust these spurious simulation trajectories despite overwhelming data against it from both experiments and other computational work.

Reviewer #2

(Remarks to the Author)
The authors have provided a comprehensive revision of the manuscript.

There are only two very minor further points:

Regarding the simulation temperature: While running the simulations at higher temperatures is indeed a useful tool for increasing the sampling in the system, it can also cause the protein to partially unfold. The additional SI Fig 5 is indeed

helpful in terms of understanding calcium and magnesium ion binding, it does not give the full picture in terms of protein stability. Could the authors add a Figure that addresses protein stability at high temperatures - eg. RMSD and/or secondary structure content over simulation time.

Could the legend in Figure 1E be shifted such that the data can be fully seen.

Reviewer #3

(Remarks to the Author)
The authors have satisfactorily answered most of my comments. However, the proposed role of PS in lipid gating is confusing. How is it possible for the charged headgroup of PS to pass through the hydrophobic BK fenestrations? What would the authors expect regarding the BK channel gating incorporated in a pure PS bilayer? The point is that if the channel gate is the lipid, one would expect a large stabilisation of the channel-open configuration, but the results of Moczydlowski et al. (1985). J. membr. Biol., show only a marginal leftward shift of the activation curve when comparing neutral PE bilayers with PS bilayers.

Reviewer #4

(Remarks to the Author)
I co-reviewed this manuscript with one of the reviewers who provided the listed reports. This is part of the Communications Chemistry initiative to facilitate training in peer review and to provide appropriate recognition for Early Career Researchers who co-review manuscripts.

Version 3:

Reviewer comments:

Reviewer #1

(Remarks to the Author)
I greatly appreciate the authors' rebuttal. Even though I remain skeptical, I will agree to disagree. The readers can draw their conclusions provided with the simulation data and the authors' presentation. Nonetheless, I do urge the authors to include an explicit statement regarding the possibility of simulation artifacts, which could due to high voltage used here, inherent limitations of the force fields, and/or possible limitations with the initial structures.

Reviewer #2

(Remarks to the Author)
The authors have sufficiently addressed the minor concerns brought in the previous review.

Reviewer #3

(Remarks to the Author)
I continue to consider the section addressing the role of PS in lipid gating as the manuscript's weakest. I recommend that the authors address this topic with greater caution and temper their conclusions regarding PS's involvement in BK channel gating. In my view, if hydrophobic gating results solely from lipid presence within the fenestrations and the channel's internal cavity, then in a pure PS bilayer, the leftward shift of the BK channel activation curve should be significantly greater than the approximately -30 mV reported by Moczydlowski et al., which corresponds to a change in free energy to open the channel of only about 1kT.
Otherwise, I think the paper is fine.

Reviewer #4

(Remarks to the Author)
I co-reviewed this manuscript with one of the reviewers who provided the listed reports. This is part of the Communications Chemistry initiative to facilitate training in peer review and to provide appropriate recognition for Early Career Researchers who co-review manuscripts.

Open Access This Peer Review File is licensed under a Creative Commons Attribution 4.0 International License, which permits use, sharing, adaptation, distribution and reproduction in any medium or format, as long as you give appropriate credit to the original author(s) and the source, provide a link to the Creative Commons license, and indicate if changes were made.
In cases where reviewers are anonymous, credit should be given to 'Anonymous Referee' and the source.
The images or other third party material in this Peer Review File are included in the article's Creative Commons license, unless indicated otherwise in a credit line to the material. If material is not included in the article's Creative Commons license and your intended use is not permitted by statutory regulation or exceeds the permitted use, you will need to obtain permission directly from the copyright holder.
To view a copy of this license, visit https://creativecommons.org/licenses/by/4.0/

Reviewer #1 (Remarks to the Author):

This is an interesting computational study of the role of lipids in gating of the BK channel. The authors have performed extensive atomistic and coarse-grained simulations of the channel in various state (Ca binding, voltage) and lipid environment (w/ and w/o POPS) to investigate the conformational dynamics of the channel and how lipid fenestration may play a central role in driving the hydrophobic gating of the channel. Even though lipid fenestration has been observed and discussed in several studies perviously, this is one of the first studies that focus on the role of lipid fenestration in BK gating. The central conclusion is that lipid fenestration is a critical determinant of BK conductivity. The authors further proposed that negatively charged lipids such as POPS could reduce lipid fenestration and promote channel activation. I think these are valuable insights that could add to the mechanistic understanding of BK channels. However, I also have several important concerns for the authors to consider.

1) It is curious and at the same time quite troubling that the channel display significant instabilities in the simulations reported in this work. This is particularly surprising and concerning in the simulation of the open, Ca2+-bound state. It is well established experimentally that the calcium bound state of the channel should remain stably open. The strange blockage of channel by lipid in Ca2+ bound and 300 mV should be considered a clear artifact of the simulation, instead of reliable refection of how the channel behave. The observation that the full lipid could enter and occupy the pore is extremely suspicious, and to me, is a sign of simulation artifacts. There is no evidence from any experiment that would support anything beyond lipid tail fenestration.

The spontaneous lipid block in some simulation replicas of the Ca^{2+}-bound state was indeed something we did not fully expect, as lipid block characterizes the closed state in our simulations. However - also prompted by the comments by other reviewers - upon reanalyzing our data, we discovered relatively frequent unbinding of Ca^{2+} from its binding sites (predominantly from the RCK1 binding site), with already one ion unbinding during 0 V simulations and more following after applying voltage. As Ca^{2+} has an activatory effect on BK, its unbinding could contribute to the lower structural stability of the channel. We now discuss these considerations in *Results/Spontaneous closure of the open state*, and provide the relevant plots on *SI Fig. 5*:

To note, spontaneous unbinding of the activatory Ca^{2+} from its CryoEM binding sites was a consistent phenomenon throughout the simulations; unbinding occurred predominantly from the RCK1 site, with one ion unbinding during equilibrium simulations with more following in applied voltage simulations (*SI Fig. 5*), which could contribute to the reduced structural stability of the Ca^{2+}-bound state.

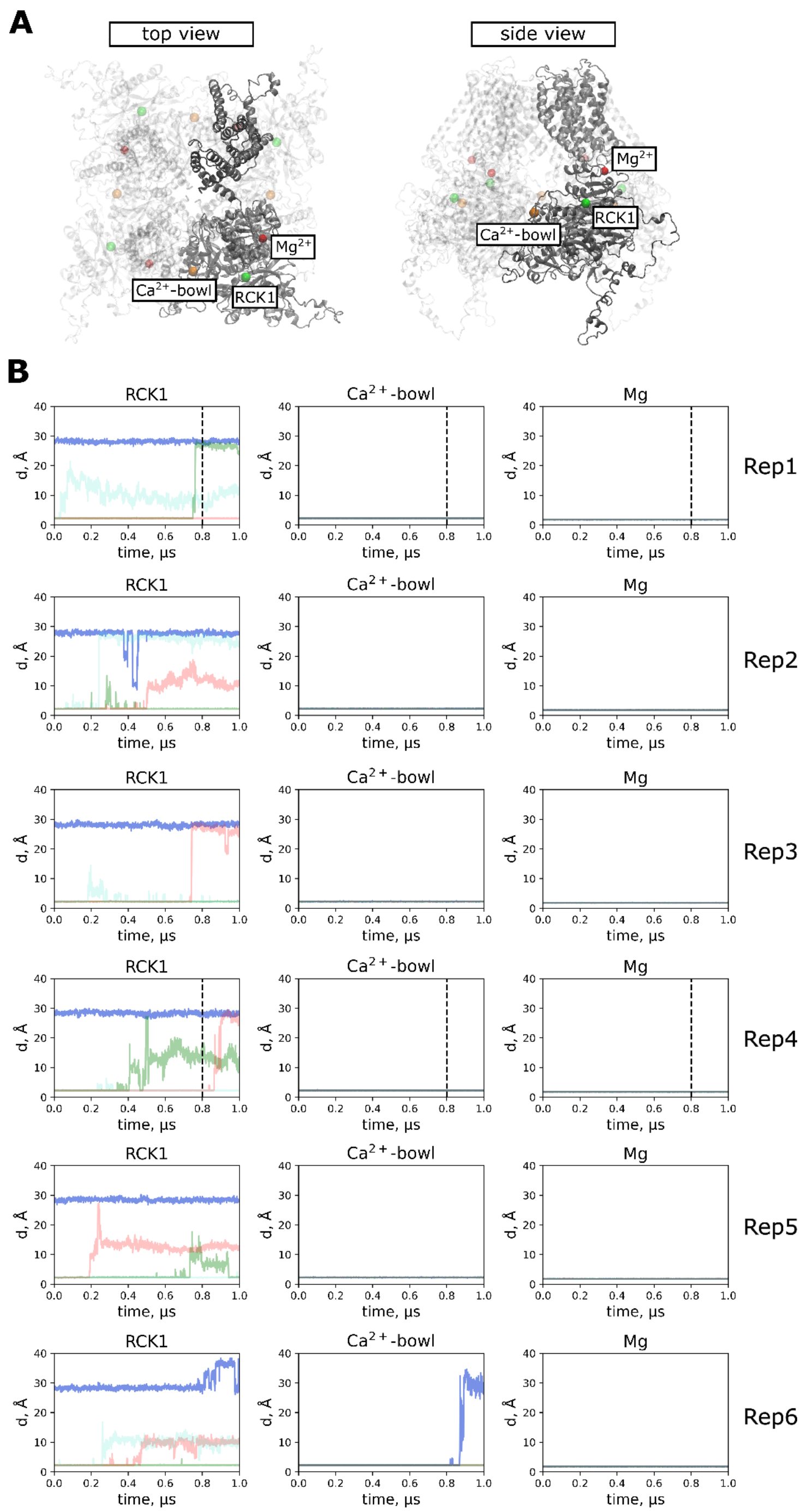

SI Fig. 5. (A) BK contains two Ca2+-binding sites - RCK1 (green) and Ca2+-bowl (orange), as well as one Mg2+-binding site (red) per subunit. The ion binding sites belonging to one of the four subunits are shown as non-transparent, and transparent for other subunits. (B) Time traces of the distance from a given binding site to the nearest Ca2+ or Mg2+ respectively, illustrating spontaneous unbinding of Ca2+ (each color corresponds to one subunit), simulations of Ca2+-bound under +300 mV. Dashed vertical lines in replicas Rep1 and Rep4 indicate time when full lipid block happened.

Taking that into account, we want to stress that the sequence of conformational changes that occurred during closure broadly agrees not only with the difference between closed- and open-state CryoEM structures, but also with the available data on the KRQK/S6 glutamate bridge formation upon channel closure that we discuss in the manuscript (*Tian et al, PNAS 2019*). We hope these conformational changes by themselves will be of use to the community, even if they aren't representative of the entire equilibrium energy landscape of the Ca^{2+}-bound state.

Considering the ability of entire lipid molecules being able to enter into the pore, in BK channels CryoEM structures so far show only lipids in the fenestrations themselves - we and other computational studies observe deeper entry of lipids already in the partial lipid block mode. However, the CryoEM structure of the TREK-1 K^+ channel showed a POPE molecule fully blocking the pore (*Schmidpeter et al, Nature Communications 2023*) - it is especially interesting as it has similar membrane-facing fenestrations lining the pore as BK channels do. Moreover, a very recent CryoEM study shows lipid densities in the pore of the TWIK-1 channel, both with lipids residing in fenestrations, as well as entire lipid molecules residing in the pore (*Ma et al., Nature Communications 2026*). Overall, this indicates that the full lipid block mode can be a valid occurrence in K^+ channels, albeit further structural studies would be needed to confirm that for BK channels experimentally. We should note that we have reworked the part where we list the available experimental evidence considering lipid block:

Generally, both mechanisms align with experimental data, including some of the CryoEM structures of BK containing lipid tails in fenestrations mentioned earlier (supporting partial block) [11], mutants of F315 (human BK, equivalent to F304 in Aplysia BK) located in fenestrations having activatory/deactivatory effect depending on their size (bigger residues activate, while smaller deactivate the channel) (Lippiat et al. 2000; Carrasquel-Ursulaez et al. 2015), possibly indicating modulation of the steric barrier for lipid entry, as well as the dependence of Po and single-channel currents on the membrane composition (such as activation of the channel by lipids with bigger headgroups, which can be explained by a higher energetic barrier for full lipid entry through the fenestrations between the S6 helices) [44,45,54]. To note, BK in the fully closed state was shown to be insensitive to the large pore blocker bbTBA (Tang et al. 2009) - while we don't preclude that there might be states where the pore is completely inaccessible to bbTBA due to sterical barrier provided by e.g. full lipid block (the pore in WT Ca^{2+}-free CryoEM states is wide enough to fit bbTBA), a recent CryoEM structure of BK with a deactivating mutation in the voltage sensing domain showed the pore narrow enough to prevent bbTBA entry, which could also explain lack of closed-state block by bbTBA (Contreras et al. 2025). At the same time, in a CryoEM structure of a K^+ channel from the K2P family (TREK-1), which has similar membrane-exposed fenestrations in the closed state, it was possible to resolve a lipid molecule occupying the pore [55]. A recent CryoEM study also showed lipid densities both in fenestrations and inside the pore of another K2P family member TWIK-1 (Ma et al. 2026), which not only confirms the results of an earlier MD study showing lipid entry into the pore in TWIK-1 [56] -, but also indicates that full lipid block can generally serve as a pore closure mechanism - albeit further structural studies would be needed to confirm that for BK channels.

2) The above examples are quite representative of the most important reservation that I have for this work. The authors seem to adopt a dangerous approach of taking everything observed in simulations at face value. Despite important advances in the reliability of atomistic simulation, this approach can easily lead to misleading and oftentimes completely wrong conclusions. Coupled with the seemingly blind trust of simulation, there are dangerous signs of confirmation bias when discussing experimental observations. I think it is crucial to take a much more critical approach in analysis and interpretation of the simulations.

We appreciate the reviewer's input on this matter. Upon rereading the manuscript & addressing all the reviewers' comments, we tried to focus on creating a balanced representation on the available data and made edits accordingly. We made a range of edits throughout the manuscript, including adding alternative interpretations (e.g. discussion of the possibility of full lipid block given the data on large pore blockers & the recent CryoEM structures that further drive the channel into a deeper closed state with a narrower pore), discussion of the channel's stability in MD as mentioned in response to the previous comment and more. We hope that together this provides a more impartial view on the subject.

3) Previous simulations by Chen and Cui labs suggest that pore dehydration does not depend on lipid fenestration, even though free simulations do observe dynamic lipid fenestration coupled with pore dehydration. This seems to argue that, despite the correlation between pore hydration and lipid fenestration, dehydration is the driving event and lipid fenestration is not. If the authors want to argue the opposite, control simulations with lipids excluded from fenestration much be performed to test if fenestration is indeed required. Importantly, the channel must be unrestrained to allow conformational response to dehydration of the pore. It is interesting to note that the Martini simulations seem to suggest that lipid fenestration can only occur if the pore is forced to dehydrate, even though that these coarse-grained simulations need to be taken with a large grain of salt.

This is indeed a great point and something we missed to indicate in the first version of the manuscript. Such simulations have already been done, recently published by another group in *Coronel et al., Nature Communications 2025*. In this work, free energy calculations were used to scan various lipid occupancies of the pore and how they affect the energy of pore dehydration - in general, the greater number of lipid atoms in the pore led to a more stable dewetted state. Importantly, they showed similar behavior not only in CHARMM, but in the Amber force field too. We now cite this paper in *Introduction*:

In several MD studies of BK channels pore dehydration was accompanied by entrance of lipid tails into the pore through the fenestrations between the pore-forming S6 helices [12,34–36], further addressed in a recent computational study that showed facilitation of pore dehydration by penetrating lipids (Coronel et al. 2025).

, and in *Discussion*:

At the same time, the pore-lining S6 helices of neighboring subunits form membrane-exposed fenestrations in Ca^{2+}-free structures (deactivating conditions), which in some CryoEM structures contain lipid tails 11, in addition to spontaneous occupancy of the pore by lipid tails in MD studies [34], shown to increase the hydrophobicity of the pore in the closed state in a recent computational study (Coronel et al. 2025).

4) I have strong reservations on the use of Martini simulations to examine detailed interactions and fenestration of lipids with ion channels. The lipid tails (and waters) are too "fat" due to the spherical representation of 4 CH2 or H2O with a single bead. Such coarse-graining severely distort the geometry fit of the tail (and water) in fenestration window and the pore. The fact that Martini simulations fail to observe pore dehydration is a clear indication of the limitation of Martini simulations in examining such detailed questions. The difference between pore occupancy is insignificant (e.g., Fig 3B) and there is great danger in interpreting these small differences.

The reduced accuracy of coarse grained force fields is indeed something that needs to be taken into account. In order to study the effect of lipid composition on BK, we used the Martini force field primarily due to the sampling limitations of atomistic simulations. With already two lipid species present, at much smaller timescales of atomistic simulations (~1 μs per replica opposed to ~40 μs that we used for Martini3 simulations) there's a risk of trapping the system in a local minimum of a certain lipid configuration, and studying the effects of that specific lipid configuration - albeit at an expense of lower accuracy of the coarse-grained simulations. As an example, both Martini and control atomistic simulations of BK in POPE:POPS showed interactions of POPS with the KRQK and its repulsion from S6Glu, which was also reflected in the reduced interactions between the KRQK & S6Glu residue clusters in the atomistic simulations, although the absolute values of contact frequencies differed between the two initial lipid distributions that we started the atomistic simulations with (*Fig. 4B, SI Fig. 10*). Another example is the local concentration of K^+ in the pore - only one simulation set (out of the two with different initial lipid configurations) had significant pore occupancy by POPS headgroups, which resulted in the increase of pore occupancy by K^+, opposite to no such effect in the other set (*SI Fig. 9*). This not only illustrates the respective strengths & weaknesses of the atomistic and coarse grained approaches (for atomistic simulations - higher resolution and the presence of conformational effects at a cost of more difficult sampling & the necessity to conduct simulations starting from more than one initial configuration), but also - despite these limitations - that the mechanistic effects of negatively charged lipids were still in a broad agreement between the Martini3 and CHARMM36m simulations.

Regarding the specific points brought up by the reviewer, the lack of pore dehydration in Martini3 is actually reflective of the behavior of the Ca^{2+}-free structure in atomistic simulations: there, the channel is initially hydrated, and moreover stays hydrated and conductive in some simulation sets (*SI Fig. 1*), similar to simulations published in *Contreras et al, Nature*

Communications 2025, which is likely a feature of the CryoEM structure. Accordingly, Martini, which keeps the structure close to the initial CryoEM conformation via elastic network, also shows a hydrated pore Furthermore, the differences in pore occupancies shown on *Fig. 3B* with and without POPS are statistically significant ($p \sim 10^{-21}$), and, mapping back from coarse-grained to atomistic resolutions would correspond to the decrease from ~12 non-hydrogen lipid atoms simultaneously residing in the pore in POPC to ~8 in POPE:POPS - in other words, by more than 30%. This is coincidentally similar to the increase of Po in POPE:POPS relative to POPE in experiments (from 0.355 to 0.544 on average, or by ~30%; *Crowley et al, Molecular Pharmacology 2005*) - such comparisons are of course not rigorous due to the different conditions used in simulations and experiments (including use of POPC instead of POPE) and the fact that we don't strictly simulate open probability that would require sampling of all possible lipid configuration & protein conformation combinations, but nevertheless suggestive that such a barrier imposed on lipid entry in case of negatively charged lipids can have an effect on the channel's activity.

Reviewer #2 (Remarks to the Author):

Paper Review

Summary
The manuscript broadly addresses BK channel opening and closing mechanisms facilitated by lipids using MD simulations to observe atom and near-atom level dynamic motions of the channel.
The authors investigate the concept of "physiological" BK closed state, which appears to be absent from experimental structures, and focus on the role of lipid tails during the pore dehydration event, previously observed in MD simulations of an isolated pore domain. The authors show that lipids are able to block the pore due to lipid tails entering fenestrations in the pore, and causing pore dehydration. The authors apply an electric field across the membrane in order to be able to classify the Ca-bound, hydrated pore structure as 'open', and the Ca-free, lipid-blocked as closed, and also classify a third partially blocked Ca-free state with intermediate conductance. The authors offer an explanation for the experimentally observed phenomenon that charged lipids play a role in regulating channel opening, by showing that PS is less likely to block the pore, and further decreases the number of potassium ions in the pore. The paper therefore addresses two separate, but related concepts – dehydration and closure of the channel correlated with lipid tail entry into the pore, and channel activation mediated by negatively charged lipid headgroups.

The introduction gives a comprehensive summary of the state-of-the-art. The authors are to be commended on the range of simulation techniques used. The findings are certainly exciting in terms of furthering our understanding of the complexity of lipid regulations of membrane protein

function. The results are compelling and justify the conclusions drawn. At times the presentation of the results could be a little clearer, with specifics detailed below.

We thank the reviewer for this positive evaluation as well as the constructive criticism.

There are a number of points:
Intro:
It would be nice to have an idea of the protein primary expression tissue and the role it has in the organism.

We have expanded the corresponding part in the *Introduction* accordingly:

Potassium (K+) channels enable permeation of K+ ions across biological membranes with high conductance and high selectivity, which plays an important role in a variety of cellular processes 1–3. In particular, BK channels ('big K+', also known as maxiK or Slo1) stand out among other K+ channels due to their particularly large single-channel conductance - 100-300 pS 4. BK channels are activated synergistically by intracellular Ca2+ and membrane depolarization; this allows them to participate in a plethora of important physiological functions across various tissues, e.g. maintaining smooth muscle arterial tone, K+ secretion in kidneys and termination of action potential in neurons (Latorre et al. 2017; Contreras et al. 2013; González-Sanabria et al. 2021).

Methods:
What is the reason for running the simulations at 323 K (50 °C)?

While we agree that 50 °C is indeed a relatively high temperature, it nevertheless helped overcome the sampling limitations and observe the functionally relevant behavior of the channel, in general agreement with experimental data. First simulations in this work were done at a relatively low temperature of 290 K (17 °C), however both the Ca^{2+}-free and Ca^{2+}-bound states behaved essentially identically in terms of having a hydrated pore and a similar low conductance, prompting us to run further simulations at a higher temperature in order to achieve better sampling of conformational transitions and ion permeation. Some effects (such as unbinding of Ca^{2+} ions in the Ca^{2+}-bound state we address in response to one of the comments below) might be considered undesirable, the channel's behavior that is potentially consequential to these effects (e.g. spontaneous lipid block of the Ca^{2+}-bound state) still aligns with previously published data (such as the differences between Ca^{2+}-bound and Ca^{2+}-free CryoEM structures and the conformational changes occurred during the spontaneous block of the Ca^{2+}-bound state in our simulations).

Results:
It would be helpful to have a short summary of the simulations performed at the start of each results section.

We describe the overall simulation scheme in the beginning of the *All-atom simulations of Ca^{2+}-bound and Ca^{2+}-free states* section:

[…] we first performed atomistic MD simulations of BK from Aplysia californica in equilibrium (i.e. at 0 mV), and then under applied voltage starting from various frames of the equilibrium runs, in both Ca2+-bound and Ca2+-free states (PDB ID 5tj6 [9] and 5tji [8], respectively; +300 mV, POPC membrane, 0.8 M KCl, CHARMM36m). The simulation durations for each condition tested are given in SI Table 1.

We don't list the simulations performed in the *Spontaneous closure of the open state* section, as this section is dedicated to simulations already described in *All-atom simulations of Ca^{2+}-bound and Ca^{2+}-free states*. We describe the coarse-grained simulations in the beginning of the *Effect of membrane composition on lipid block* section:

[...] we first performed coarse-grained simulations of Ca2+-bound and Ca2+-free states of BK using the Martini3 force field [46] in a POPE:POPS (3:1 mol.) membrane - the ratio used in the experimental study [42] - with control simulations in a POPC membrane (*SI Table 2*). The switch from all-atom to coarse-grained simulations was done to increase the accessible simulation times and extensively sample the relevant configurations of lipids around the protein. After 40 μs-long simulations [...]

We also decided to split the *SI Table 1* into *SI Table 1* (with simulations from the first two *Results* subsections), and *SI Table 1* (with simulations from the *Effect of membrane composition on lipid block* subsection of *Results*) for easier referencing of these simulations.

Since the control atomistic simulations of BK in POPE:POPS are mentioned only at the end of *Effect of membrane composition on lipid block*, we briefly mention them there, now with the corresponding sentences slightly rephrased:

[...] in our control atomistic simulations of BK in POPE:POPS (*SI Table 1*), we observed a higher frequency of states with larger KRQK-glutamate distances in POPE:POPS compared to the simulations in POPC [...] Additionally, we performed atomistic simulations of an isolated pore domain in POPE:POPS and POPC as an approach to increase sampling due to a largely reduced size of the system (*SI Table 2*).

The use of the word 'replica' is a little confusing, as it might mislead the reader to think that the simulations were run in the replica-exchange regime. The authors might consider using the word 'repeat' instead.

We understand how this confusion might possibly arise and appreciate the reviewer's input to prevent reader's confusion, although we should note that the term 'simulation replica' is a common way to refer to simulation repeats, including cases where no sampling enhancement is

used (such as replica exchange simulations), as for example used in https://elifesciences.org/reviewed-preprints/88403, https://www.nature.com/articles/s41467-025-61638-9, https://www.nature.com/articles/s41467-023-37531-8. To avoid such possible confusion, we defined 'simulation replica' upon its first mentioning:

The pore of Ca2+-bound stayed hydrated throughout most of the equilibrium and applied voltage simulations in agreement with previous studies (Fig. 1B), with the exception of a decrease in pore hydration in one equilibrium replica (an independent repeat of simulations) out of 5 [...]

Section on all-atom simulations of calcium-bound and calcium-free states: According to Table S1, calcium-bound and calcium-free states with applied voltage were run with 6 repeats. Why can we only see three repeats for each setup in Fig 1C, D.

If the reviewer meant SI Fig. 1C, D (the supplementary figure), then Ca^{2+}-bound indeed was run with 6 repeats at +300 mV; however, as the Ca^{2+}-free structure showed varied behavior at 0 V (hydrated pore, partial lipid block or full lipid block), for Ca^{2+}-free we initiated applied voltage simulations using a frame for each of the states of the pore correspondingly We list these simulations in SI Table 1 for the combination Ca^{2+}-free/POPC/CHARMM36m/+300mV:

3x0.2 μs (started from a 0 mV replica with no lipid block), 6x0.6..0.7 μs (started from two 0 mV replicas with partial lipid block with 3 applied voltage replicas for each initial frame), 4x1 μs (started from a 0 mV replica with full lipid block)

SI Fig. 1C, D thus shows lipid/water/ion traces for the repeats of Ca^{2+}-free where the pore remained hydrated (3 repeats), or only partially blocked by lipids (3 repeats for each of the two initial frames, 6 repeats in total).

Might the authors consider specifically referring to which repeat is being discussed, eg: as int the ms:"spontaneous dehydration events under applied voltage (2 out of 6 replicas), and a decrease in pore hydration in one equilibrium replica out of 5 (Fig. 1B, SI Fig. 1B)". It is not clear which of the repeats is the single spontaneous dehydration event (end of Rep2?)

We have now specified the replicas shown on the figures that we refer to throughout the text, as well as the description of SI Fig. 1B.

And also here: from the ms: "the Ca2+-free channel showed a dehydrated pore in all simulations except 1) one equilibrium replica out of five". In Fig 1A it looks like rep 1 and rep5 are dehydrated – could this be clarified?

Both 'Rep1' and 'Rep5' on SI Fig. 1A indeed show pore dehydration and partial lipid block. However, in this sentence we denote the replica where pore dehydration didn't happen, which corresponds only to the replica 'Rep4'. Prompted by this comment, as well as a comment by

another reviewer we now mention an approximate hydration level above which the channel is conductive in *Results/All-atom simulations of Ca^{2+}-bound and Ca^{2+}-free states*:

In terms of pore hydration, consistent presence of at least 20 water molecules in the pore (using our definition of the pore region; see Methods) appeared to correlate with a conductive channel.

“in the simulations of the Ca2+-free state at +300 mV where the pore remained hydrated and not blocked by lipids, the channel showed steady outward K+ permeation”

Could the conductance of Ca-free, fully hydrated at +300mV be added as a third bar in Fig 1D?

We now added the corresponding conductance values to Fig. 1D.

In the CHARMM36m simulations of the Ca-bound, did the eight calcium ions remain bound over the course of the simulation?

Ca^{2+} ions did indeed unbind in the simulations of the Ca^{2+}-bound state, which might have contributed to the spontaneous closure of the Ca^{2+}-bound state, albeit we couldn’t tell the exact patterns of Ca^{2+} becoming unbound that led to this closure in some replicas but not others. We added figure with time traces of Ca^{2+}-binding site occupancies in *SI Fig. 5*:

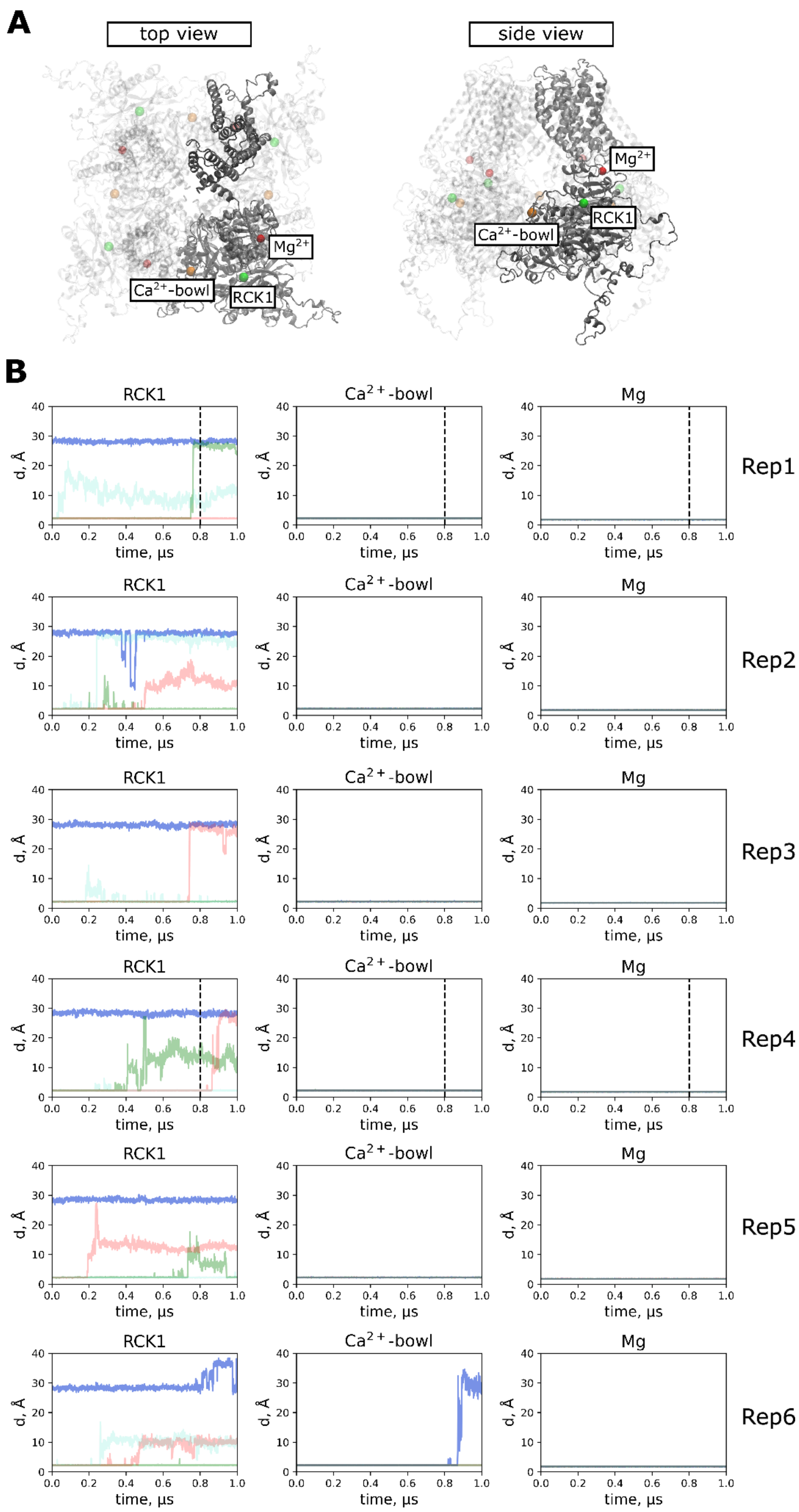

SI Fig. 5. (A) BK contains two Ca2+-binding sites - RCK1 (green) and Ca2+-bowl (orange), as well as one Mg2+-binding site (red) per subunit. The ion binding sites belonging to one of the four subunits are shown as non-transparent, and transparent for other subunits. (B) Time traces of the distance from a given binding site to the nearest Ca2+ or Mg2+ respectively, illustrating spontaneous unbinding of Ca2+ (each color corresponds to one subunit), simulations of Ca2+-bound under +300 mV. Dashed vertical lines in replicas Rep1 and Rep4 indicate time when full lipid block happened.

as well as a discussion of this effect in *Results/Spontaneous closure of the open state*:

To note, spontaneous unbinding of the activatory Ca^{2+} from its CryoEM binding sites was a consistent phenomenon throughout the simulations; unbinding occurred predominantly from the RCK1 site, with one ion unbinding during equilibrium simulations with more following in applied voltage simulations (*SI Fig. 5*). We don't preclude that this could contribute to the spontaneous closure via the conformational changes described below, although other reasons (e.g. the employed Ca^{2+}-bound CryoEM structure being a metastable state in simulation conditions) are possible as well.

Discussion:
Could the authors comment on how the inclusion of cholesterol might have altered the results, since this is a major component of the plasma membrane. Was there a rationale to not including cholesterol?

Cholesterol was shown to have an (inhibitory) effect on BK activity [https://link.springer.com/chapter/10.1007/978-3-031-21547-6_8]. However, while the interplay of the effects of various lipids on BK in membranes with complex compositions is an interesting question, we focused specifically on the mechanism of activation by negatively charged lipids. In this case using a simpler two-component membrane (POPE:POPS) - which, importantly, showed a significant increase relative to membranes with no charged lipids in experiments [https://molpharm.aspetjournals.org/content/68/1/4] - allowed us to isolate the effect of specifically negatively charged lipids.

Could the authors comment on how the choice of atomistic force field might have affected the results (eg CHARMM36m vs AMBER)? A paper from 2020 (https://doi.org/10.1021/acs.jctc.0c00331) reports notable differences in pore stability in Charmm36 vs. Amber simulations of potassium channels.

The majority of simulations of BK gating-related processes were done using the CHARMM36m force field, which we also used in our study in order to align with previous findings. However, in a recent work by Coronel et al, 2025 (https://www.nature.com/articles/s41467-025-61638-9) both CHARMM36m and Amber ff15ipq/LIPID17 were used to study the effect of lipid occupancy of the pore on the dehydration free energy. The results from both force fields were in a broad agreement. We now cite this paper in *Introduction*:

In several MD studies of BK channels pore dehydration was accompanied by entrance of lipid tails into the pore through the fenestrations between the pore-forming S6 helices [12,34–36], further addressed in a recent computational study that showed facilitation of pore dehydration by penetrating lipids (Coronel et al. 2025).

, and in *Discussion*:

At the same time, the pore-lining S6 helices of neighboring subunits form membrane-exposed fenestrations in Ca^{2+}-free structures (deactivating conditions), which in some CryoEM structures contain lipid tails 11, in addition to spontaneous occupancy of the pore by lipid tails in MD studies [34], shown to increase the hydrophobicity of the pore in the closed state in a recent computational study (Coronel et al. 2025).

In the Martini3 simulations, the dehydrated pore has, on average 25 lipid beads in the pore, which corresponds roughly to 75-100 non-H atoms (although difficult to be sure of this conversion without knowing the Martini bead types). This is higher than the number of lipid atoms in the pore in the Charmm36m simulations (Fig1B, Fig 2A from 800-1000 ns). Could the authors justify this?

The pore of the Ca^{2+}-free state underwent little dehydration in the Martini3 simulations, unless water was expelled from the pore using restraints; in the manuscript, apart from other potential biases of the Martini force field, we link this to the elastic network used in Martini which preserves the initial, hydrophilic pore conformation of the Ca^{2+}-free CryoEM structure. Thus, in order to estimate the differences in lipid entry when the pore is already dehydrated, we decided to use the approach of expelling water molecules from the pore with restraints - opposed to a more natural dehydration seen in atomistic simulations, this might bias not only hydration levels of the pore, but the overall lipid occupancy as well (again, not taking the other force field differences between Martini3 and CHARMM36m). To note, we now describe the restraint scheme in more detail in the *Methods* section:

In order to prevent water beads from entering the pore in the respective simulations, a spherical flat-bottomed repulsive position restraint with a radius of 1.1 nm, centered on the center of mass of I301 backbone beads was used; initially, the pore contained no water beads.

Figures:
Overall, the figures could be made more helpful in terms of understanding the results presented by the authors. In their current state, the figures are disjointed, require repeated and laborious cross-referencing with the supplementary figures, and are not self-sufficient, but instead rely heavily on the explanations for the main text. For example:

The overview of the structure presented in Figure 1A is useful and an excellent inclusion, and it would be useful to zoom in on the pore area, to show this in clearer detail.

We added a picture of the pore domain to Fig. 1A.

Fig 1B: it is not clear if these graphs show data from all simulations, or for a single repeat. This could be made more explicit.

We edited the figure legend which now reads as follows:

Distribution of the number of lipid atoms (non-hydrogen atoms out of ~50 per lipid molecule), and water molecules in the pore over all replicas in the all-atom simulations of Ca^{2+}-free and Ca^{2+}-bound without applied voltage.

It would be helpful if eg helix S6 were labelled in Fig 1C

We labeled S6 on Fig. 1C.

Fig 1E, S2a,C: what is the reason for using radians? Degrees would be perhaps more conventional.

We changed units to degrees in Fig. 1E and SI Fig. 2A, C.

Fig2C, D: the structure representation could perhaps be included to make clearer, eg render the helices with a less transparent material. It would also be helpful to note in the figure legend that F304 is coloured red.

Rendering the entire protein unfortunately makes understanding the motion of lipids visually more difficult. However, in combination with S6 helices labeled on Fig. 1C, as per one of the previous comments by the reviewer, we hope the overall indication of the direction of motion of the lipid from the membrane into the pore through the pore helices is made more clear. Additionally, we now note in the legend of Fig. 2C,D the color of F304 on the structure.

Fig. 3D only makes sense when compared to uncharged lipid densities (Fig. S7A), so both should be presented in the same figure.

We moved the panel with POPE densities (Ca^{2+}-free) from SI Fig. 7A to Fig. 3D.

It should be made more explicit in the figure legend why 1/3 of POPE is being shown in Fig. 4B.

We added a more detailed explanation to the legend of Fig. 4B.

Reviewer #3 (Remarks to the Author):

Based on detailed molecular simulations, the present manuscript strongly argues in favor of lipid gating of BK channels. Previous work, including studies by one of the authors of the present manuscript, has been in favor of BK channel closure mediated by a hydrophobic gating mechanism. This type of gating was mediated by a decrease in the internal cavity diameter and

an increase in its hydrophobicity, resulting from the rotation of the S6 transmembrane segment and the exposure of amino acid residues F304 and I308 to the cavity. The authors now contest that even the narrowest internal BK cavity of a closed BK channel reported in a recent paper (Contreras et al. 2025. Nature Communications) is not narrow enough to hinder K+ conduction. Thus, to promote pore dehydration and close the channel, lipid must enter through the channel fenestrations present only in closed BK channels. Given the unrestricted conditions for lipid movement through the fenestrations during the simulations, the results presented are convincing and show that a lipid block can be partial or, if the lipid occupies the internal cavity, complete. This observation leads to interesting consequences regarding the dwell time of the closed states. Of note is the finding that pore dehydration was always accompanied by the entrance of lipids to the pore. The authors further determine the ability of the BK channel to conduct ions by performing simulations in the presence of a 300 mV applied voltage. The effect of membrane lipid composition was also studied, concluding that lipid pore occupancy is reduced when the membrane composition is a mixture of neutral and negatively-charged lipids (POPE:POPS).

Comments
1. The paper by Coronel et al. (2024) not only suggested the necessity of lipid entry for pore dehydration, but their findings also led them to the conclusion that gating was controlled by lipid entrance through the lateral fenestrations. Even the title of that paper is not very different from that of the manuscript by Minorenko et al. The authors cannot downplay the importance of this previous work. They should establish in the discussion the novelty of their study and the contribution to a further understanding of BK lipid gating.

We thank the reviewer for the suggestion and apologize for omitting this work. Coronel et al. indeed provide valuable evidence on how lipid occupancy of the pore affects the pore's propensity for dewetting. Notably, they see a similar effect in both CHARMM and Amber force fields. On the other hand, we directly show how lipid entry affects conductance using simulations under applied voltage, focus on the interplay between conformational changes and ability of the channel to conduct ions, and, importantly, the effect of negatively charged lipids on the channel's functional state.

We now cite this paper in *Introduction*:

In several MD studies of BK channels pore dehydration was accompanied by entrance of lipid tails into the pore through the fenestrations between the pore-forming S6 helices [12,34–36], further addressed in a recent computational study that showed facilitation of pore dehydration by penetrating lipids (Coronel et al. 2025).

, and in *Discussion*:

At the same time, the pore-lining S6 helices of neighboring subunits form membrane-exposed fenestrations in Ca^{2+}-free structures (deactivating conditions), which in some CryoEM structures contain lipid tails 11, in addition to spontaneous occupancy of the pore by lipid tails in MD

studies [34], shown to increase the hydrophobicity of the pore in the closed state in a recent computational study (Coronel et al. 2025).

We have additionally slightly rephrased several points throughout the manuscript to account for the positive evidence for lipid mediation of pore dehydration that this study provides.

2. The sentence in the Introduction, "In a recent CryoEM study, mutations of voltage-sensing arginines that promote deactivation made it possible to obtain structures with the pore as narrow as ~8 Å in diameter, which nevertheless can still fit a hydrated K+ ion" is misleading. The evidence suggest that a pore ~8 Å in diameter can be dehydrated if it is hydrophobic enough. Given the structural results of Contreras et al. (2025), which show that the internal vestibule becomes quite hydrophobic upon channel closure, the importance of pore diameter and hydrophobicity should be discussed in more detail.

We see how this sentence might be confusing - here, we only meant that in the CryoEM structures that were obtained in deactivating conditions the pore is still large enough so that movement of a hydrated K^+ ion is sterically possible - however, later we mention that one possible barrier to ion flow in the close state is pore dewetting. We have now rephrased the sentence to hopefully prevent possible confusion:

In a recent CryoEM study, mutations of voltage-sensing arginines that promote deactivation made it possible to obtain structures with the pore as narrow as ~8 Å in diameter [12], which is nevertheless wide enough that it's sterically possible to can still fit a hydrated K+ ion [15].

We should note that in Contreras et al, the pore of R3Q apo structure, while somewhat less than the WT structure, was still hydrated in MD when it was restrained to the initial (CryoEM) conformation (see Fig. 3D in Contreras et al.). Compared to model nanopores such as those described in *Paulo et al., Nat Commun 2023*, this indicates a complex wetting behavior that might be not only affected by radius (& possibly voltage), but also the hydrophobicity - which is in turn affected by pore conformation and the presence of lipids in the pore. We decided to discuss this in conjunction with electrowetting; please see the response to the next suggestion for the quote from the text updated accordingly.

3. One of the novelties of the present study is the simulations in the presence of an applied voltage to determine ion conduction. However, Paulo et al. (Nature Communications 2023) discussed the effect of applied voltages on nanopore hydration and tested their predictions by engineering a biological nanopore. It would increase the importance of the author´s findings if they confronted their results with those of Paulo et al.

We thank the reviewer for this interesting suggestion. Similar effects can indeed be found in biological pores - an example of this is *Jensen et al., PNAS 2010* (https://doi.org/10.1073/pnas.0911691107), where negative voltages induced a hydrophobic pore collapse in the Kv1.2 channel, possibly indicating the presence of voltage-dependent

wetting/dewetting in K^+ channels. In case of BK channels, one could expect the presence of lipids in the cavity to lead to a higher barrier for electrowetting, although simulations would be needed for a more concrete answer. Taking this and the work by Paulo et al. into account - and including the effects of pore radius & hydrophobicity mentioned in the previous suggestion - we added the following to *Introduction* and *Discussion*:

Notably, pore wetting has been studied for model non-biological nanopores, where the hydration state of the pore appears to depend on its radius and geometry; moreover, for a given pore that is dehydrated at 0 V, wetting can be induced by applied voltage (Paulo et al. 2023). Such voltage-dependent behavior was observed in K+ channels as well, such as the negative hydrophobic pore collapse in the Kv1.2 channel induced by negative voltages (Jensen et al. 2010). While a scan of various voltages and their effect on the dynamics of lipids/water in the BK channel's pore is beyond the scope of this work, this presents an interesting direction for future research, especially considering the conformational flexibility of BK's pore and lipids contributing to pore hydrophobicity.

4. In the Introduction and Discussion, the paper by the Lingle´s group is cited, pointing out that large organic blockers cannot block fully closed channels . The authors should be aware that Contreras et al. (2025) found, using the structure of a "fully closed" BK channel (R3Q), that hindrance to blockade is due to a decreased pore diameter. The closure at I308 is sufficient to occlude entry of bbTBA into the R3Q apo channel.

We thank the reviewer for pointing out this observation. We think this adds to the discussion regarding the existence of full lipid block: the constriction at I308 as seen in the R3Q apo structure can indeed explain the lack of block by bbTBA in the fully closed state (although not strictly precluding states with a wider pore blocked by lipids, where the lipids themselves provide the steric barrier for bbTBA entry). We edited the respective parts of *Introduction* and *Discussion* to reflect this:

To note, BK in the fully closed state was shown to be insensitive to a large pore blocker bbTBA (*Tang et al. 2009*) - while we don't preclude that there might be states where the pore is completely inaccessible to bbTBA due to sterical barrier of e.g. full lipid block (the pore in WT Ca2+-free CryoEM states is wide enough to fit bbTBA), a recent CryoEM structure of BK with a deactivating mutation in the VSD showed the pore narrow enough to prevent bbTBA entry, which could also explain lack of closed-state block by bbTBA (*Contreras et al. 2025*).

5. The authors' simulations, conducted in the presence of a 300 mV applied voltage, demonstrate a full lipid block. However, at 300 mV and in the absence of Ca2+, the probability of opening is essentially 1. How do they explain this discrepancy between the simulation and the electrophysiological result? According to the simulations, one would expect a much lower open probability.

This is a valid concern; we deem the explanation to be in the timescales on which open probability (and accordingly transitions between open & closed states) is measured in experiments (seconds), which is beyond the timescales mostly achievable by atomistic molecular dynamics simulations (microseconds). An example of successful observation of a closed-to-open-state transition is *Jensen et al., Science 2012*, where the transition of the Kv1.2/Kv2.1 channel from an initially deactivated state to a state with all voltage-sensing domains active and pore conductive was observed - albeit only after very extensive simulations of tens of microseconds under applied voltage. Accordingly, if simulations are started from a closed-state structure, one would expect the protein to behave mostly as a closed-state channel at the ~1 µs timescales and below.

6. The finding of a lower single-channel conductance in the Ca2+-free than in the Ca2+-bound condition is also at odds with the experimental result that shows that the BK single-channel conductance in the absence of Ca2+ is not different from that in the presence of the divalent cation (see Taladucker and Aldrich, Biophys J. 2000).

Similar to the comment #5 by the reviewer, the reason for a lower conductance in the simulations of Ca^{2+}-free where no lipid block happened is likely the time scales on which the channel explores all the relevant conformations at high enough positive voltages in experiments: there, the channel samples the open state, however due to the smaller timescales accessible to simulations - if these are initiated from the Ca^{2+}-free structure - the channel resides in the closed state regardless of voltage. However, it should be stressed that despite these limitations, it was nevertheless possible to observe the functional states of the channel - ability of the channel to conduct ions - with various degrees of pore hydration and lipid block. We now mention this discrepancy and its explanation in *Results/All-atom simulations of Ca^{2+}-bound and Ca^{2+}-free states:*

Notably, in those simulations of the Ca2+-free state at +300 mV where the pore remained hydrated and not blocked by lipids, the channel showed steady outward K+ permeation of 8±3 pS (Fig. 1D, SI Fig. 1C, D, SI Table 1). In contrast to this observation, experiments show similar single-channel conductances of BK in both presence and absence of Ca2+ (Talukder and Aldrich 2000). This discrepancy could be explained by the channel primarily exploring the conformations close to the initial structure (i.e. mostly functionally closed when initiated from the Ca2+-free, and mostly functionally open when initiated from the Ca2+-bound structure) on the MD timescales.

A note for the authors regarding the effect of charged lipids.
The results obtained in the presence of negatively charged lipids are exciting. They may solve an old conundrum regarding the effect of charged lipids on the probability of opening (Po) and the channel conductance. Note that Moczydlowski et al. (1985, J.Membr. Biol.) determined the effect of the charge of the lipid (PS)on the BK single-channel conductance and Po. They concluded that the surface potential generated by the charged bilayer was responsible for the effects found on channel conductance and Po. However, Modzydlowski et al. (2003, J. Gen. Physiol.) found PS lipid bilayers were able to modulate channel conductance but not external

Ba2+ and TEA blockade. Thus, the surface potential of the bilayer induced by the negative charge of the bilayer cannot explain these results.
My point here is that the results presented in this paper provide a parsimonious explanation for these contradictory results. If PS can reach the internal cavity, it should affect conductance and blockade by internal Ba2+.

We thank the reviewer for pointing it out. POPS does reach the pore in our simulations (e.g. see Fig. 4A, middle that shows an increase in the pore occupancy by negatively charged beads), with some effect on the K^+ concentration, specifically in the 'intermediate closed' hydrated Ca^{2+}-free state that allows easier entry for lipid headgroups due to wider fenestrations. The fact that Ba^{2+} block is barely affected in *Park et al, JGP 2003* (apart from the slight enhancement of block at a low salt concentration) in this case could be speculatively explained by the increased local K^+ concentration in the pore cancelling out the additional negative electrostatic potential provided by the POPS groups, thus not affecting Ba^{2+} block. We added this to *Discussion*:

In addition, an earlier experimental study showed POPS-containing bilayers altering the channel's conductance, but not Ba2+ and tetraethylammonium block (*Park et al. 2003*): this could be explained by the additional negative electrostatic potential provided by POPS being cancelled out by a locally increased K+ concentration in the pore.

Minor
1. The descriptions of the figures are confusing. For example, it is unclear why the authors started describing Fig. 1B instead of Fig.. 1A. Although it is true that in the calcium-free condition the pore is dehydrated in replicas 1 and 5, it contains a considerable number of water molecules in 3 and 4. So the question is how the authors define a dehydrated non-conductive pore. Considering that Fig. 1C and SI Fig. 3 show that a channel with 40 or less water atoms is conductive. Please clarify this point.

Regarding Fig. 1A, we mention it before Fig. 1B in *Introduction*:

The structures revealed that BK channels, in addition to the pore domain, contain the regulatory transmembrane voltage and intracellular Ca^{2+}-sensing domains that enable the synergistic activation (*Fig. 1A*).

Regarding the point about the number of water molecules, this is indeed something that we missed to indicate - SI Fig. 1A shows the entire simulation replicas of Ca^{2+}-free at 0V, which were initially hydrated & not blocked by lipids, and only later underwent pore dehydration with either partial or full lipid block. In general, using our definition of the pore (cylinder with a 10 Å radius and 20 Å depth relative to the center of mass of T276), having consistently more than 20 water molecules in the pore seems to correlate with a conductive channel. We added the following to *Results/All-atom simulations of Ca^{2+}-bound and Ca^{2+}-free states*:

In terms of pore hydration, consistent presence of at least 20 water molecules in the pore (using our definition of the pore region; see *Methods*) appeared to correlate with a conductive channel.

2. Please indicate in the text to which replicas you are referring, as in many cases, it is simply stating “the replica shows” without a number.

We have edited the text and the figure descriptions to make for a more clear referencing of replicas.

3. The ordinate given in number of atoms is confusing. For example, if, as the legend says, "Times traces of the number of lipid (non-hydrogen) atoms, the reader would like to know how many lipids the number of atoms represents.

We now specified the total number of non-hydrogen atoms per lipid (~50) correspondingly across the manuscript.

4. Fig. 2A and SF Fig. 3 top show the same replicas. This is confusing.

The rationale of including all replicas of Ca^{2+}-bound on SI Fig. 3 was to show both the lipid-blocked and the non-lipid-blocked replicas on one figure for easier comparison, as well as to have a consistent plot structure and numbering of replicas throughout SI Fig. 3 and SI Fig. 4. Per previous suggestion, we now also specify the replica numbers as shown on the figures that we refer to throughout the text; we hope it will also resolve potential confusion here.

5. For consistency, the authors should use either the number of atoms or the number of water molecules in the internal cavity (cf. SI Fig. 1 and Fig. SI Fig. 3).

We now switched to using water molecules instead of water oxygen atoms, and changed the SI Fig. 1 and its caption accordingly.

Reviewer #4 (Remarks to the Author):

I co-reviewed this manuscript with one of the reviewers who provided the listed reports. This is part of the Communications Chemistry initiative to facilitate training in peer review and to provide appropriate recognition for Early Career Researchers who co-review manuscripts.

Reviewer #1 (Remarks to the Author):

The authors' strong efforts to address my previous comments are greatly appreciated. Many of the changes made have improved the manuscript. However, my following major reservations remain.

1) The authors stated that full lipid molecules have been observed in the pore K2P channels (Ma et al, 2026). I actually could not find such data/conclusion in my reading of the same paper. Even if what the authors stated is true, K2P is quite different from BK. None of ~10 BK structures from several independent labs has ever observed densities that could suggest a full lipid in the pore. There is also minimal lipid density (or fenestration window) in the open state structures. The experimental evidence is missing to support the possibility of full lipid occupancy in the pore; instead, all evidence supports that only lipid tails can enter the pore transiently.

The CryoEM structures with lipids resolved in the pore of TWIK-2 can be seen on Fig. 2B, E in Ma et al., Nat. Commun. 2026. Another recent work by Mondal et al., Nat Commun 2026 presents a new set of CryoEM structures of TWIK-2 being fully blocked by lipids, in a manner similar to what we observe in our simulations.

We would like to stress that we observed all modes of lipid block only in the closed state of the channel: the simulations where the open state underwent lipid block were accompanied by conformational changes strongly indicative of a transition to the closed state; hence, we are not proposing lipid block in the open state of the channel.

Whether the data on K2P channels is transferrable to BK channels is a legitimate question. We would argue that qualitatively, there is a strong resemblance of the overall closure mechanisms between these K2P and BK channels - in particular dehydration of the pore, and lipid tails entering into the pore through the side openings between the pore-lining helices. We agree that ideally, in order to definitively confirm (or deny) the existence of a full block in BK channels, more high-resolution structures of the closed-state BK, where lipids are generally resolved, are needed. In this work, we aimed to present the observations from our simulations, and reconcile them with the existing experimental data in a hopefully balanced manner. We provide an overview of the experimental data regarding modes of lipid block in *Discussion*:

Both modes of lipid block were able to stop ion permeation on MD timescales (microseconds). Generally, both mechanisms align with experimental data, including some of the CryoEM structures of BK containing lipid tails in fenestrations mentioned earlier (supporting partial block) [11], mutants of F315 (human BK, equivalent to F304 in Aplysia BK) located in fenestrations having activatory/deactivatory effect depending on their size (bigger residues activate, while smaller deactivate the channel) [58,59], possibly indicating modulation of the steric barrier for lipid entry, as well as the dependence of Po and single-channel currents on the membrane composition (such as activation of the channel by lipids with bigger headgroups, which can be explained by a higher energetic barrier for full lipid entry through the fenestrations between the S6 helices) [47,48,60]. To note, BK in the fully closed state was shown to be insensitive to the large

pore blocker bbTBA [19] - while we don't preclude that there might be states where the pore is completely inaccessible to bbTBA due to sterical barrier provided by e.g. full lipid block (the pore in WT Ca^{2+}-free CryoEM states is wide enough to fit bbTBA), a recent CryoEM structure of BK with a deactivating mutation in the voltage sensing domain showed the pore narrow enough to prevent bbTBA entry, which could also explain lack of closed-state block by bbTBA [46]. At the same time, in a CryoEM structure of a K^+ channel from the K2P family (TREK-1), which has similar membrane-exposed fenestrations in the closed state, it was possible to resolve a lipid molecule occupying the pore [61], notably confirming the results of an earlier MD study (Jorgensen et al. 2016). Recent CryoEM studies also showed lipid densities both in fenestrations and inside the pore of another K2P family member TWIK-2 (Ma et al. 2026; Mondal et al. 2026).Taken together, these results indicate that full lipid block can generally serve as a pore closure mechanism - albeit further structural studies would be needed to experimentally test this mechanism for BK channels.

2) It is interesting that the authors discovered that Ca2+ was lost in some of the simulations of the open state w/ Ca2+ bound and 300 mV, which may explain instabilities in these trajectories. However, Ca2+ binding to CTD should be very tight and the observation that Ca2+ was lost itself is a huge red flag (and not reported by any of the published atomistic simulations). My honest take is that many things could go wrong and lead to "surprising" and often confusing observations in atomistic simulations. Most of the time, these are due to technical reasons (such as inadequate equilibration, artifacts in the initial structure and numerous other possibilities). I think it is dangerous to trust these spurious simulation trajectories despite overwhelming data against it from both experiments and other computational work.

We appreciate the reviewer's concerns regarding this matter; we would like to address these concerns as follows:

1. Force field treatment of Ca^{2+} binding is a well-documented challenge for additive force fields. The direction and magnitude of force field errors for Ca^{2+} binding are highly context-dependent (*Tan et al., ACS Phys Chem Au 2021; Li et al., JPCB 2015*). While the standard model overestimates binding in many contexts (particularly to carboxylate-rich coordination sites), the absence of polarization effects can also lead to an incorrect balance of interactions that destabilizes binding in others, especially under conditions not represented in the force field parameterization, such as the application of a strong transmembrane electric field. We therefore emphasize that the Ca^{2+} unbinding we observe reflects a known force field inaccuracy for divalent cation–protein interactions, rather than a simulation-specific technical failure.

2. The reviewer notes that Ca^{2+} unbinding has not been reported in published BK simulations. To our knowledge, none of the published studies, where the full-length channel in the open, Ca^{2+}/Mg^{2+}-bound state is used, actually demonstrate the behavior of Ca^{2+} ions throughout the simulations (Coronel et al., Nat. Commun 2025; Zhang et al., Nat Commun 2022; Tian et al., PNAS 2019; Yazdani et al., eLife 2020). Strictly speaking, this is not indicative that Ca^{2+} remains bound in these simulations.

3. An important factor that has been overlooked in this discussion is the applied transmembrane voltage of +300 mV. Consistent with a voltage-dependent contribution to unbinding, our data show that Ca^{2+} unbinding was more pronounced in the +300 mV simulations than in the equilibrium (0 mV) simulations (SI Fig. 5), where typically only one Ca^{2+} ion was lost during the equilibration phase compared to progressive unbinding of additional ions under applied voltage.

4. Despite the force field limitations, the pattern of Ca^{2+} unbinding in our simulations is consistent with the known biochemistry of BK Ca^{2+} binding sites:
   a. **Unbinding occurred predominantly from the RCK1 site** (SI Fig. 5), which experimentally has a lower Ca^{2+} affinity than the Ca^{2+} bowl (RCK2; Zhang et al., PNAS 2010). If the unbinding were a random numerical artifact, there would be no reason for it to preferentially affect the lower-affinity site. This selectivity argues that the force field, while inaccurate in absolute terms, captures the relative energetic ranking of the two sites.
   b. **Mg^{2+} ions remained largely stable in their binding sites throughout the simulations** (SI Fig. 5), consistent with their distinct coordination chemistry and the generally tighter Mg^{2+} binding in the CHARMM force field.

5. The spontaneous closure events are internally self-consistent with experimental structures. This is the central argument for the physical relevance of the trajectories in question. The conformational changes observed during the spontaneous closure of the Ca^{2+}-bound state: fenestration widening between S6 helices, motions of F307 and I308, and in one replica, clear salt bridge formation between E310/E313 and ^{317}KRQK^{320} closely reproduce the specific structural differences between the Ca^{2+}-bound and Ca^{2+}-free cryoEM structures. These are not generic unfolding events or random conformational drift. If these trajectories were dominated by force field artifacts or technical problems, there is no reason why the resulting conformational changes would recapitulate the specific, independently determined structural features of channel closure. The probability of reproducing multiple correlated structural signatures of a known functional transition by random chance is small. We therefore maintain that these trajectories capture physically relevant dynamics, even if the timescale is influenced by premature Ca^{2+} loss.

6. The core conclusions do not depend on the Ca^{2+}-bound closure events. We wish to emphasize that the central claims of our manuscript, that lipid block is a defining feature of the non-conductive state, and that negatively charged lipids activate BK through a multi-modal mechanism are primarily established by the Ca^{2+}-free simulations and the membrane composition analysis (Figs. 1, 3, 4), which are entirely independent of the Ca^{2+} unbinding issue. The spontaneous closure events in the Ca^{2+}-bound state (Fig. 2) provide complementary, supporting evidence for the mechanism, but are not the sole or even primary basis for any of our main conclusions.

We have now updated the section *Spontaneous closure of the open state* in *Results* to include a more detailed discussion of Ca^{2+} unbinding in the context of the force field accuracy, simulation conditions and experimentally measured affinities:

As we mentioned earlier, the Ca^{2+}-bound state remained hydrated and conductive throughout most of our simulations; however, in two out of six replicas at +300 mV the pore of the open channel eventually underwent spontaneous dehydration, accompanied by a full lipid block of the pore in both cases (Fig. 2A; all Ca^{2+}-bound +300 mV replicas including those where lipid block didn't happen are shown on SI Fig. 3 for comparison) - in addition to one equilibrium replica with decreased pore hydration and increased lipid presence in the pore (SI Fig. 1B, 'rep2'). To note, spontaneous unbinding of the activatory Ca^{2+} from its CryoEM binding sites was a consistent phenomenon throughout the simulations. Unbinding occurred predominantly from the RCK1 site, with one ion unbinding during equilibrium simulations with more following in applied voltage simulations (SI Fig. 5), which could contribute to the reduced structural stability of the Ca^{2+}-bound state. Non-polarizable force fields tend to generally overestimate binding affinities of divalent cations (Li et al. 2015; Tan et al. 2021); in our case, the application of a relatively strong transmembrane electric field might have contributed to a lower apparent binding affinity of Ca^{2+} to its binding sites. The pattern of unbinding also reflects the experimentally suggested relative binding affinities of various sites, with stronger binding to the 'Ca^{2+}-bowl' site compared to RCK1 (Zhang et al. 2010).

Reviewer #2 (Remarks to the Author):

The authors have provided a comprehensive revision of the manuscript.

There are only two very minor further points:

Regarding the simulation temperature: While running the simulations at higher temperatures is indeed a useful tool for increasing the sampling in the system, it can also cause the protein to partially unfold. The additional SI Fig 5 is indeed helpful in terms of understanding calcium and magnesium ion binding, it does not give the full picture in terms of protein stability. Could the authors add a Figure that addresses protein stability at high temperatures - eg. RMSD and/or secondary structure content over simulation time.

We added a figure with plots showing conservation of the protein's secondary structure post equilibration (*SI Fig. 13*):

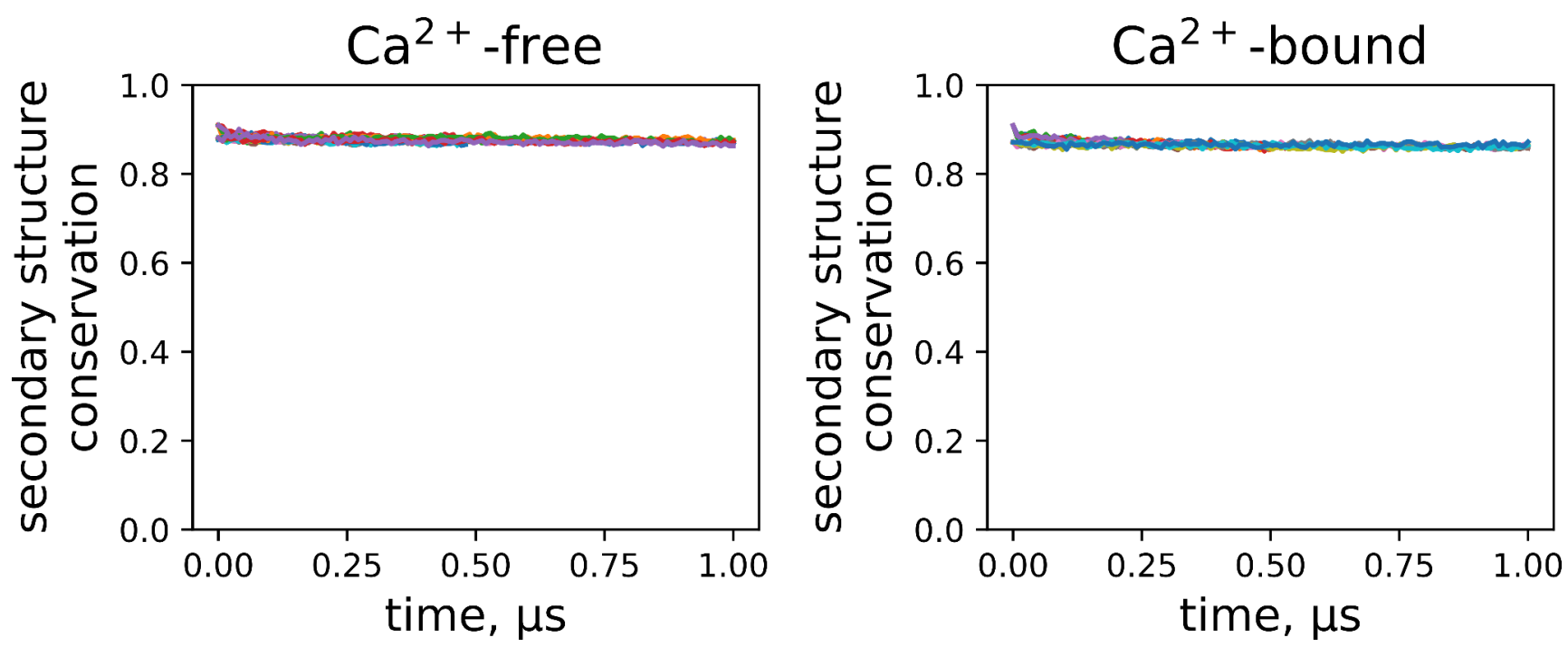

SI Fig. 13. Fraction of the residues with conserved secondary structure relative to Ca^{2+}-bound and Ca^{2+}-free CryoEM structures, respectively, as a general measure of BK conformational stability under simulation conditions. Different line colors correspond to different simulation conditions; all simulations performed are shown.

Could the legend in Figure 1E be shifted such that the data can be fully seen.

We have now rearranged Fig. 1E in order to make the data more visible:

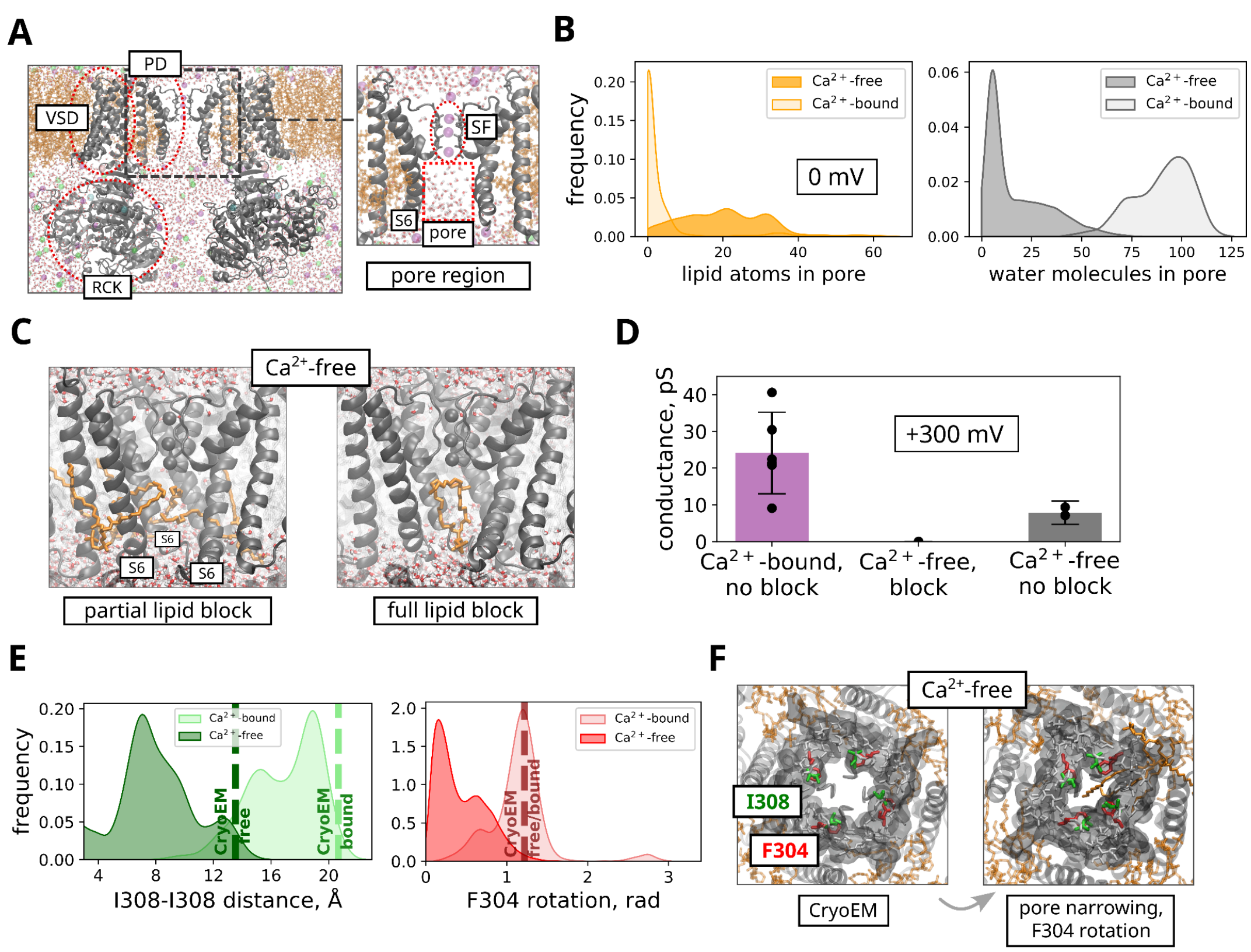

Reviewer #3 (Remarks to the Author):

The authors have satisfactorily answered most of my comments. However, the proposed role of PS in lipid gating is confusing. How is it possible for the charged headgroup of PS to pass through the hydrophobic BK fenestrations? What would the authors expect regarding the BK channel gating incorporated in a pure PS bilayer? The point is that if the channel gate is the lipid, one would expect a large stabilisation of the channel-open configuration, but the results of Moczydlowski et al. (1985). J. membr. Biol., show only a marginal leftward shift of the activation curve when comparing neutral PE bilayers with PS bilayers.

Considering the ability of charged PS headgroups to pass through the inter-helix fenestrations: generally, we observed lower frequency of PS lipids entering the pore, which we link to the negative electrostatic potential provided by the E310/E313 residues surrounding the pore (Fig. 3). However, when it happens, the occasional crossing of the hydrophilic PS headgroups (and lipid headgroups in general) into the pore seems to be possible due to the headgroups being generally located below the part of the pore that undergoes dehydration, i.e. in a hydrophilic environment - where they are also interacting with the positively charged KRQK cluster on the pore domain/RCK linker, and are able to coordinate K^+ (Fig. 4).

We thank the reviewer for pointing us to the results of Moczydlowski et al. (J. Membr. Biol., 1985). Generally, the experimental results we have cited in our manuscript so far were obtained in mixed membranes (i.e. charged and neutral lipids at the same time); we took the same approach in our study in order to align with these works. However, Moczydlowski et al. show generally the same trend in the channel's behavior when switching from POPE to POPS - increased Po and single channel currents. Quantitatively, the effects appear to be significant: increase of unit conductance from ~240 to ~340 pS (0.1 M KCl; Fig. 2 in Moczydlowski et al.), and increase of Po from ~0.5 to >0.9 (40 mV, 0.2 μM Ca^{2+}; Fig. 4 in Moczydlowski et al.). As for the mechanistic effects of pure PS bilayers on an atomistic level, one could carefully speculate that there might be certain peculiarities of the channel's behavior in pure PS opposed to 25% PS in our case, for example different dynamics of the KRQK-E310/E313 salt bridge formation that seem to be affected by negatively charged lipids in our case, or off-pore effects, such as voltage sensor dynamics, where we also observe some binding of PS in our simulations. However, in our opinion substantial simulation studies would be needed to make more concrete claims regarding BK behavior in pure PS bilayers.

We now cite Moczydlowski et al. when discussing experimental results on activation by negatively charged lipids:

Finally, we investigated the mechanism of BK activation by negatively charged lipids. It has been shown that such lipids can increase both the Po of the channel, as well as single-channel current (Vaithianathan et al. 2008; Crowley et al. 2005; Moczydlowski et al. 1985).

Reviewer #4 (Remarks to the Author):

I co-reviewed this manuscript with one of the reviewers who provided the listed reports. This is part of the Communications Chemistry initiative to facilitate training in peer review and to provide appropriate recognition for Early Career Researchers who co-review manuscripts.

Reviewer #1 (Remarks to the Author):

I greatly appreciate the authors' rebuttal. Even though I remain skeptical, I will agree to disagree. The readers can draw their conclusions provided with the simulation data and the authors' presentation. Nonetheless, I do urge the authors to include an explicit statement regarding the possibility of simulation artifacts, which could due to high voltage used here, inherent limitations of the force fields, and/or possible limitations with the initial structures.

We thank the reviewer for the constructive feedback throughout the review process, and we agree to make this explicit. While we had noted several of these limitations individually, a single, consolidated statement is clearer for the reader, and we have now added the following to the *Spontaneous closure of the open state* section:

Finally, in order to summarize these results, we should note that while we did observe a concerted transition of the channel's pore towards a closed state preceded by unbinding of Ca^{2+} - overall in agreement with the experimental data - we cannot fully exclude that the spontaneous transitions observed here are influenced to some degree by limitations of atomistic simulations, including the elevated transmembrane voltage used to accelerate sampling, known inaccuracies of non-polarizable force fields (particularly for divalent cations) [47,50], and potential imperfections in atomic position in the initial CryoEM-derived structures. Therefore, all the observations from our trajectories should be interpreted with these caveats in mind.

Reviewer #2 (Remarks to the Author):

The authors have sufficiently addressed the minor concerns brought in the previous review.

We thank the reviewer for the constructive criticism in the previous rounds of revision.

Reviewer #3 (Remarks to the Author):

I continue to consider the section addressing the role of PS in lipid gating as the manuscript's weakest. I recommend that the authors address this topic with greater caution and temper their conclusions regarding PS's involvement in BK channel gating. In my view, if hydrophobic gating results solely from lipid presence within the fenestrations and the channel's internal cavity, then in a pure PS bilayer, the leftward shift of the BK channel activation curve should be significantly greater than the approximately -30 mV reported by Moczydlowski et al., which corresponds to a change in free energy to open the channel of only about 1kT.
Otherwise, I think the paper is fine.

We appreciate the reviewer's concerns on this matter. However, we would like to point out that a shift in $V_{1/2}$ of -30 mV, with 2.4*e* of BK's gating charge (Yang *et al.*, Front Physiol 2015), yields

~2.8kT of free energy difference, which is already significant - and supports the assumption that the proposed mechanism should result in significant effects on the channel's gating in experiments. Additionally, as we mentioned in our previous response, when switching to a pure PS membrane, an increase in Po from 0.5 to 0.9 was observed (40 mV, 0.2 μM Ca^{2+}; Fig. 4 in Moczydlowski et al.), as well as an increase in current from ~240 to ~340 pS (0.1 M KCl; Fig. 2 in Moczydlowski et al.), which further suggests a strong functional effect of PS lipids. We do agree however that the relative contributions of three modes we described for POPS remain unknown. We have now addressed it by adding the following sentence to *Discussion*:

We note, however, that the relative contribution of each of the three modes we described for POPS remains to be quantified, and disentangling them, for instance in membranes of varying PS content (Moczydlowski et al. 1985), is an important direction for future work.
