# Supplementary material for: Lipid gating of BK channels and activation mechanism by negatively charged lipids
Source: Commun Chem. 2026 Oct 6;9:315. doi: 10.1038/s42004-026-02245-7 (PMC13642915; doi:10.1038/s42004-026-02245-7)
Supplement: Supplementary file 2 [file 42004_2026_2245_MOESM2_ESM.pdf]

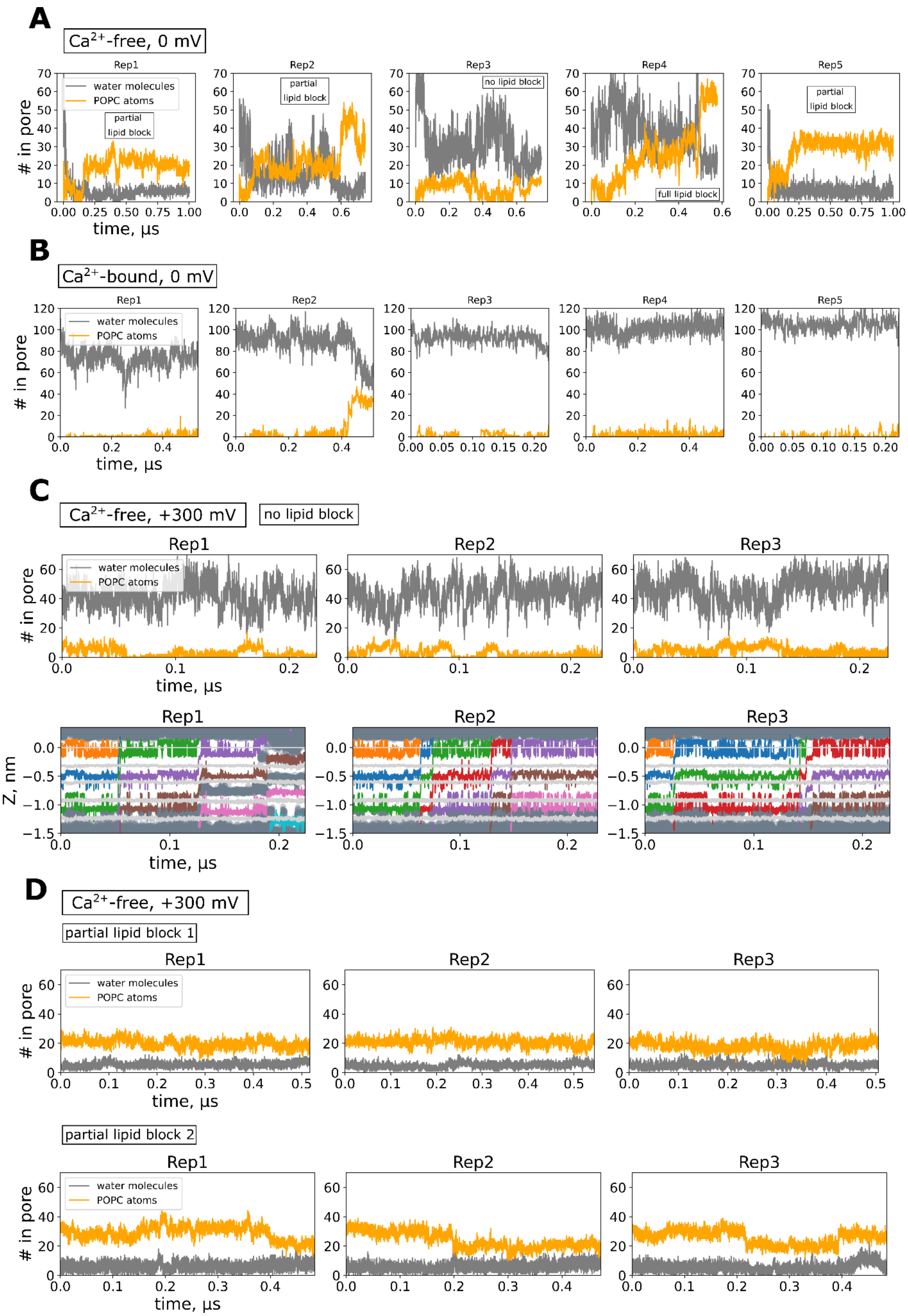
A
Ca2+-free, 0 mV
Rep1
Rep2
Rep3
Rep4
Rep5
water molecules
POPC atoms
partial lipid block
no lipid block
full lipid block
in pore
time, µs
B
Ca2+-bound, 0 mV
C
Ca2+-free, +300 mV
no lipid block
Z, nm
D
Ca2+-free, +300 mV
partial lipid block 1
partial lipid block 2

SI Fig. 1. Time traces of the number of lipid atoms (non-hydrogen atoms only; each lipid contains ~50 non-hydrogen atoms) and water molecules in the pore in atomistic simulations without applied voltage of (*A*) Ca^{2+}-free, where the pore was mostly dehydrated and blocked by lipids, except for one replica ('Rep4') which showed no lipid block and an overall higher level of hydration; (*B*) Ca^{2+}-bound, where the pore was mostly hydrated, except for one replica with some decrease in pore hydration ('Rep4'), and (*C, upper*) replicas of Ca^{2+}-free under applied voltage, where the pore remained hydrated, not blocked by lipids, and conductive, as indicated by time traces of K^+ (colored lines), water (dark gray scatter) in the SF (carbonyls and hydroxyls of SF-forming K^+ binding sites are shown as light gray lines) (*C, lower*). (*D*) Time traces of lipid and water oxygen atoms in the pore, Ca^{2+}-free +300 mV simulation sets with partial lipid block.

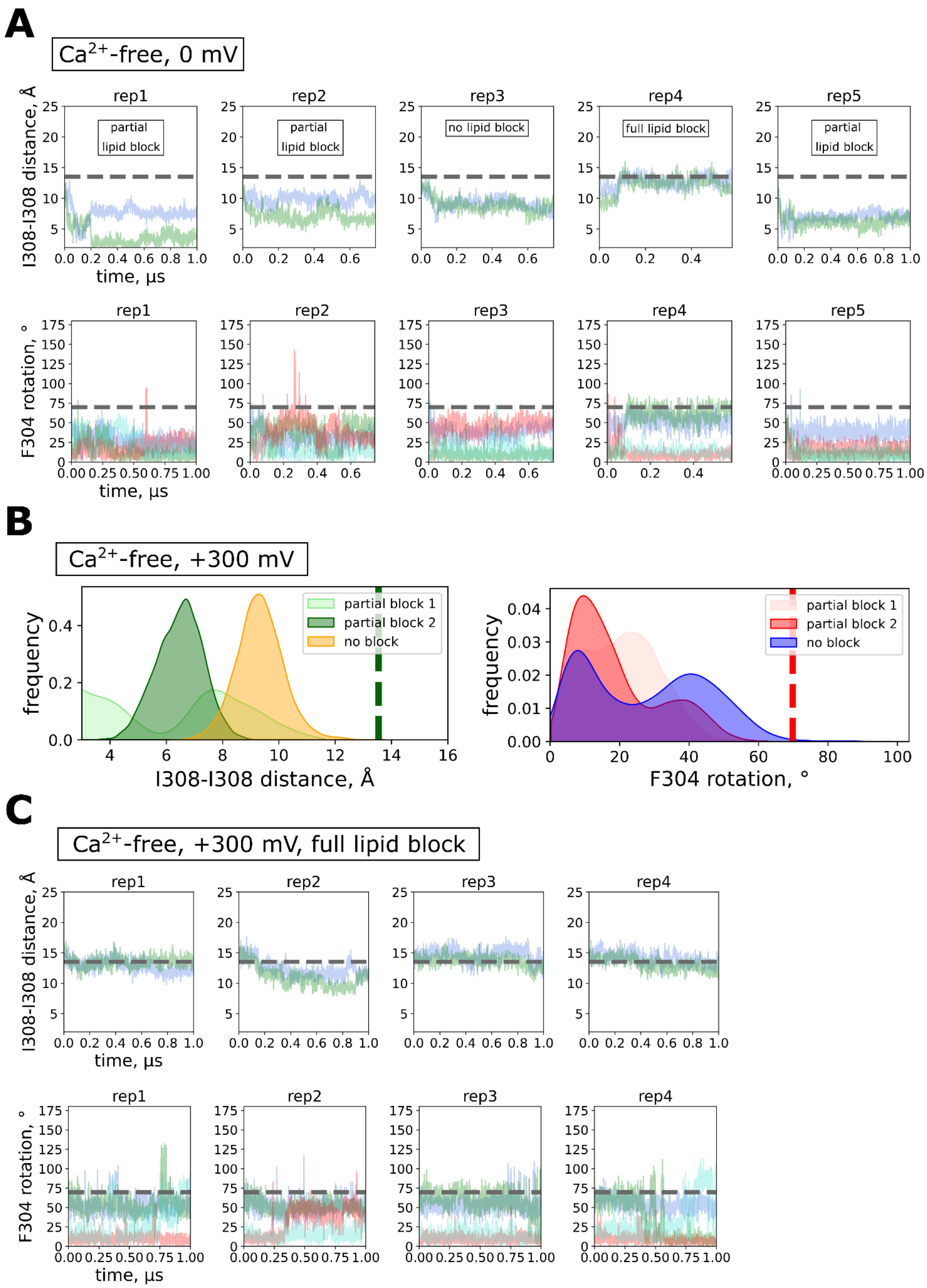

SI Fig. 2. (*A*) Time traces of pore opening (distance between CA atoms of I308 residues of opposing subunits) and inward rotation of F304 (defined as the angle between center of mass of all F304 and CA, CZ atoms of F304 for each subunit) in the Ca^{2+}-free state, in atomistic simulations without applied voltage. (*B*) Pore closure and F304 rotation in +300 mV simulations of Ca^{2+}-free in the two simulation sets with a partial lipid block, and the simulation set where the

lipid block did not occur. (*C*) Time traces of pore opening and inward rotation of F304 in the Ca^{2+}-free state, in atomistic simulations at +300 mV where full lipid block occurred. Dashed lines represent the CryoEM structure values for the Ca^{2+}-free state.

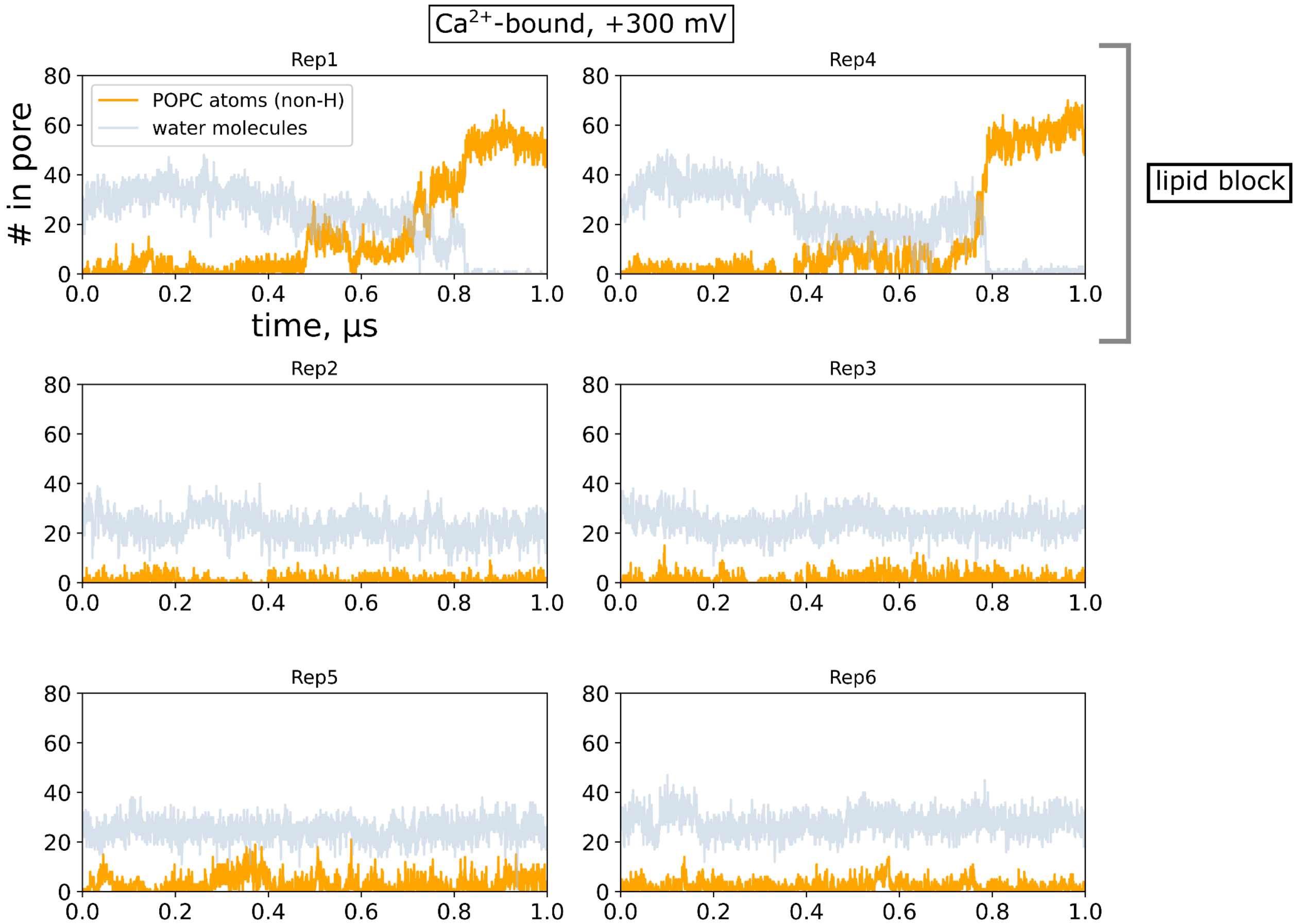

SI Fig. 3. Traces of the number of water molecules and POPC atoms (non-hydrogen, out of ~50 per lipid molecule) in the pore in the atomistic simulations of the Ca^{2+}-bound state at +300 mV. The replicas where the lipid block of the pore occurred are highlighted.

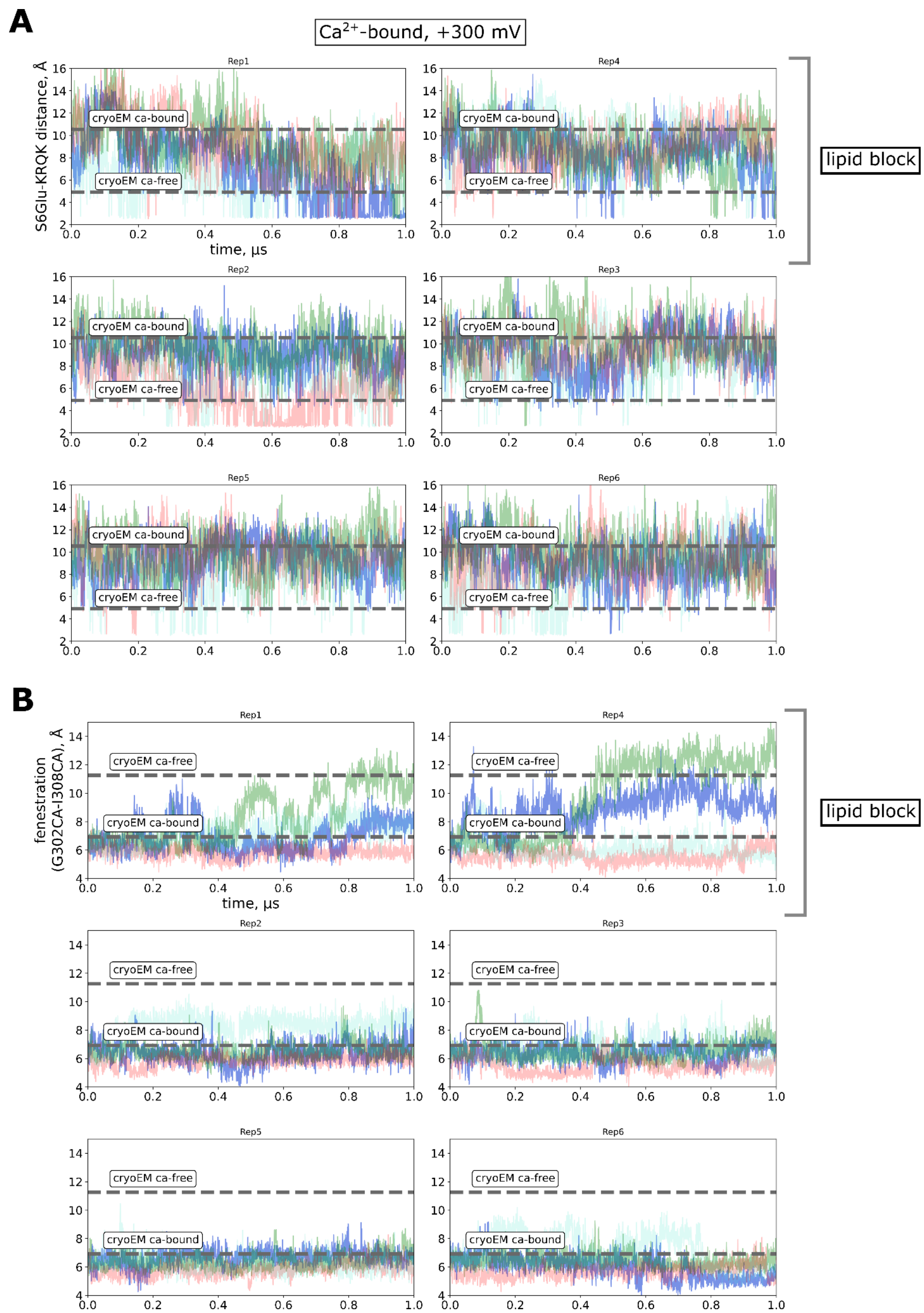

SI Fig. 4. Atomistic simulations of the Ca^{2+}-bound state at +300 mV. Time traces of (*A*) distances between E310/E313 ('S6Glu') and ^{317}KRQK^{320} of neighboring subunits, illustrating the salt bridge formation between the two charged clusters at the end of S6 helices, and (*B*) distances between CA atoms of G302 and I308 of neighboring subunits, illustrating the widening of fenestrations between S6 helices; different colors represent distances between different residue pairs (4 in total). Simulation replicas where spontaneous lipid block occurred ('Rep1' and 'Rep4') are indicated.

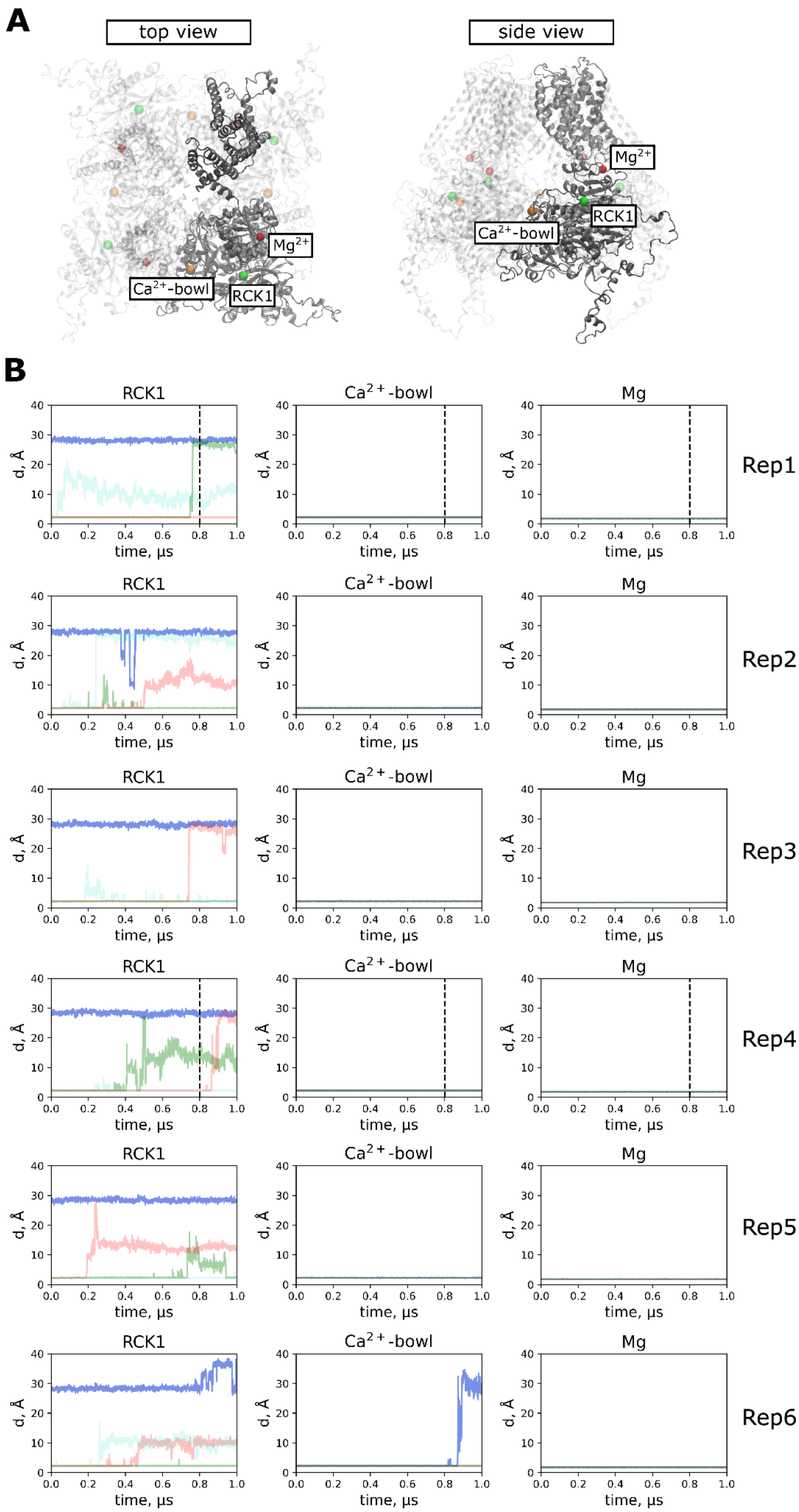
A
top view
side view
Mg2+
Ca2+-bowl
RCK1
B
RCK1
Ca2+-bowl
Mg
d, Å
time, µs
Rep1
Rep2
Rep3
Rep4
Rep5
Rep6

SI Fig. 5. (*A*) BK contains two Ca^{2+}-binding sites - RCK1 (green) and Ca^{2+}-bowl (orange), as well as one Mg^{2+}-binding site (red) per subunit. The ion binding sites belonging to one of the four subunits are shown as non-transparent, and transparent for other subunits. (*B*) Time traces of the distance from a given binding site to the nearest Ca^{2+} or Mg^{2+} respectively, illustrating spontaneous unbinding of Ca^{2+} (each color corresponds to one subunit), simulations of Ca^{2+}-bound under +300 mV. Dashed vertical lines in replicas Rep1 and Rep4 indicate time when full lipid block happened.

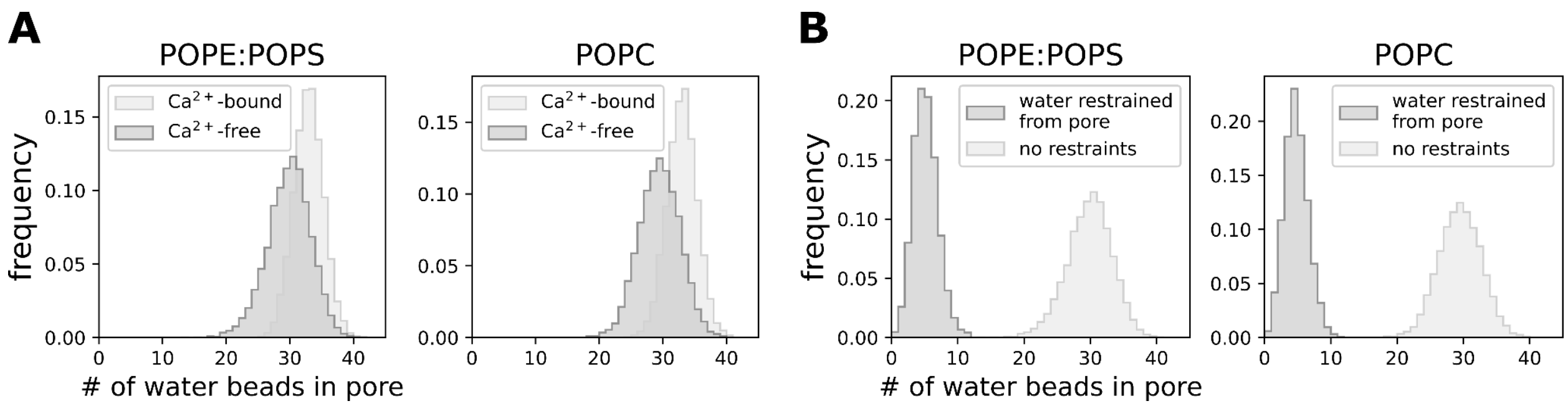

SI Fig. 6. Distribution of water beads in the pore (*A*) without, and (*B*) with restraints on water preventing it from entering the pore in Martini3 simulations.

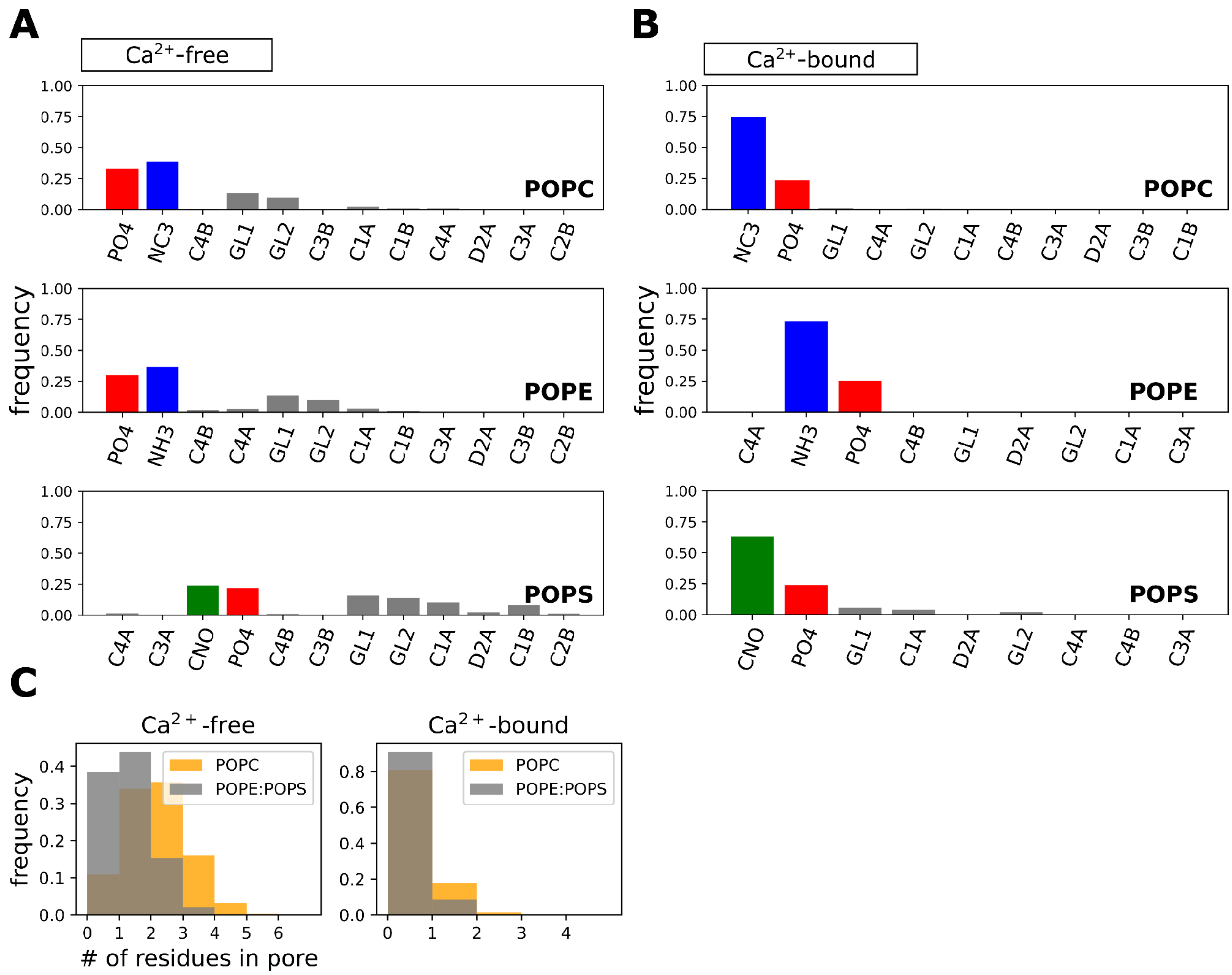

SI Fig. 7. Relative frequencies of lipid beads occupying the pore in (*A*) Ca^{2+}-free (hydrated pore) and (*B*) Ca^{2+}-bound states in Martini3 simulations; colored bars represent the headgroup beads (excluding the glycerol beads), blue corresponds to positively charged beads, red to negative and green to non-charged. (*C*) Distribution of the number of individual lipid residues simultaneously occupying the pore in Martini3 simulations.

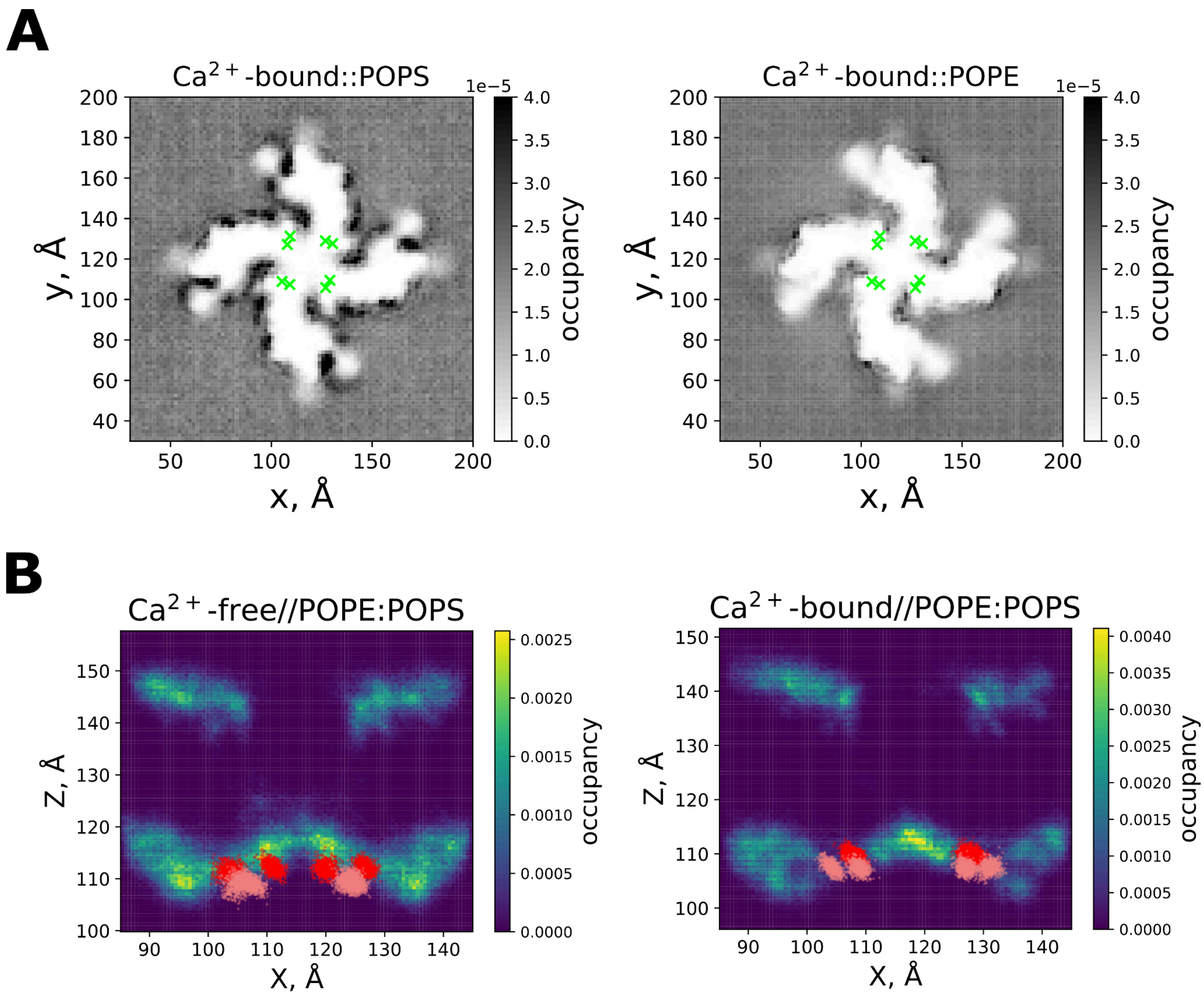

SI Fig. 8. (*A*) Lipid distribution around the channel in Martini3 simulations (Ca^{2+}-bound state); E310/E313 residues in S6 helices are shown as reference (green markers). (*B*) Occupancies of PO4 beads (corresponding to phosphate groups; only beads within 3 nm of E310/E313 in the membrane plane were counted), shown in a plane perpendicular to the membrane. Note that the densities are integrated along the Y axis, and the density in the pore region of the channel (X = 110..120 Å) does not necessarily represent PO4 beads occupying the pore. E310 is depicted as red scatter, E313 as pink scatter (all subunits).

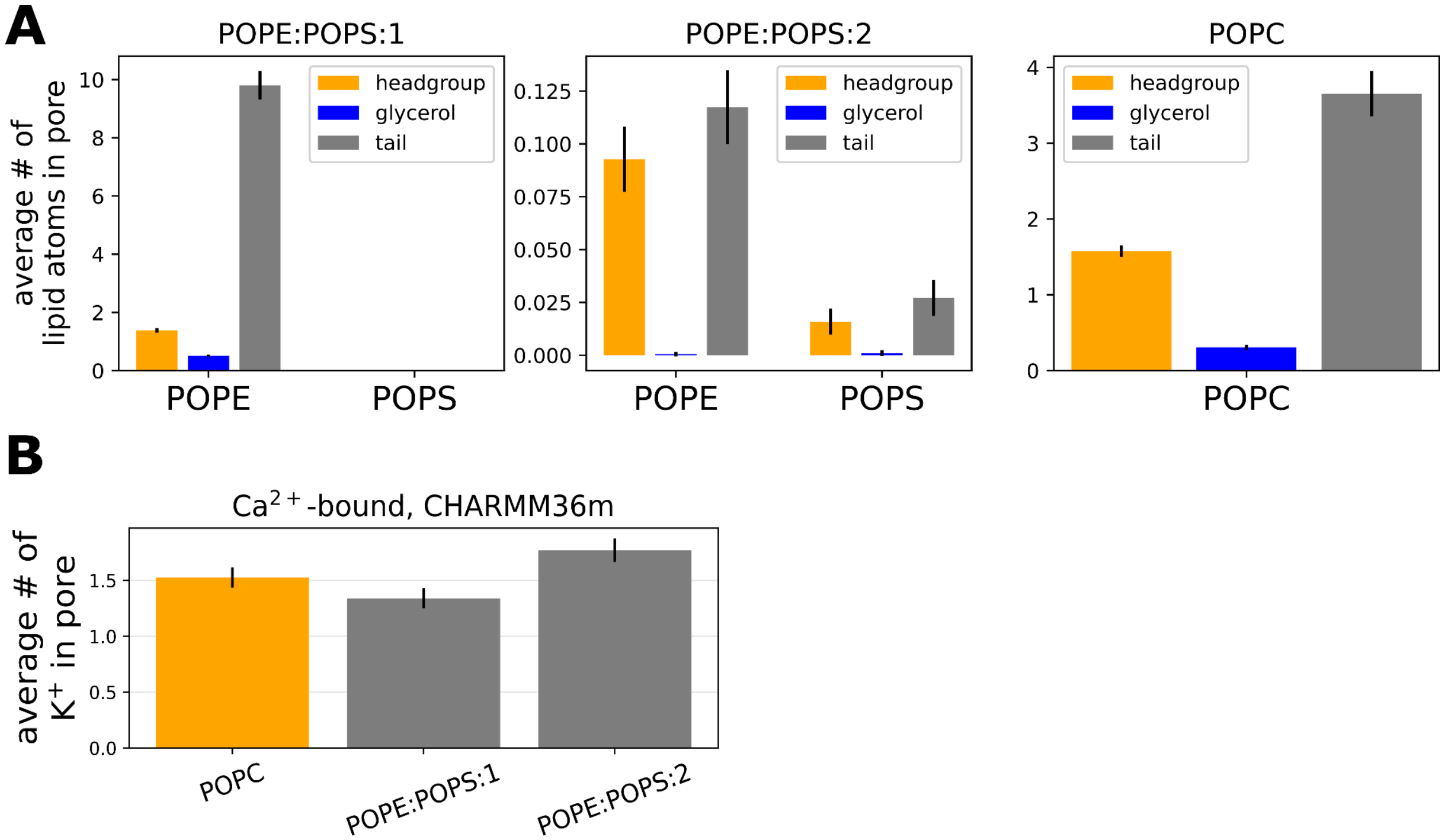

SI Fig. 9. (*A*) Occupancy of the pore by atoms belonging to either the headgroup (excluding glycerol), glycerol or the tail of a given lipid, and (*B*) average number of K^+ ions in the pore in atomistic simulations (Ca^{2+}-bound state, 0 mV).

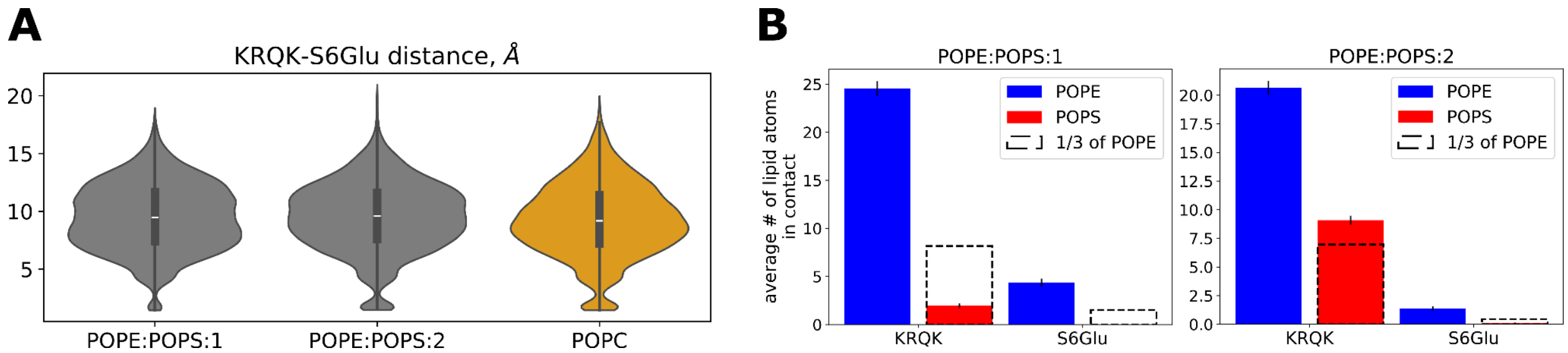

SI Fig. 10. Atomistic simulations of the full-length channel (Ca^{2+}-bound state) in POPC compared to POPE:POPS membranes. (*A*) Distributions of distances between ^{317}KRQK^{320} and E310/E313 ('S6Glu') residues of neighboring subunits. (*B*) Average number of contacts between headgroups belonging to a given lipid type, and ^{317}KRQK^{320} or S6Glu in atomistic simulations of Ca^{2+}-bound in POPE:POPS. POPE:POPS:1 and POPE:POPS:2 indicate simulations started from the two different initial configurations of the membrane.

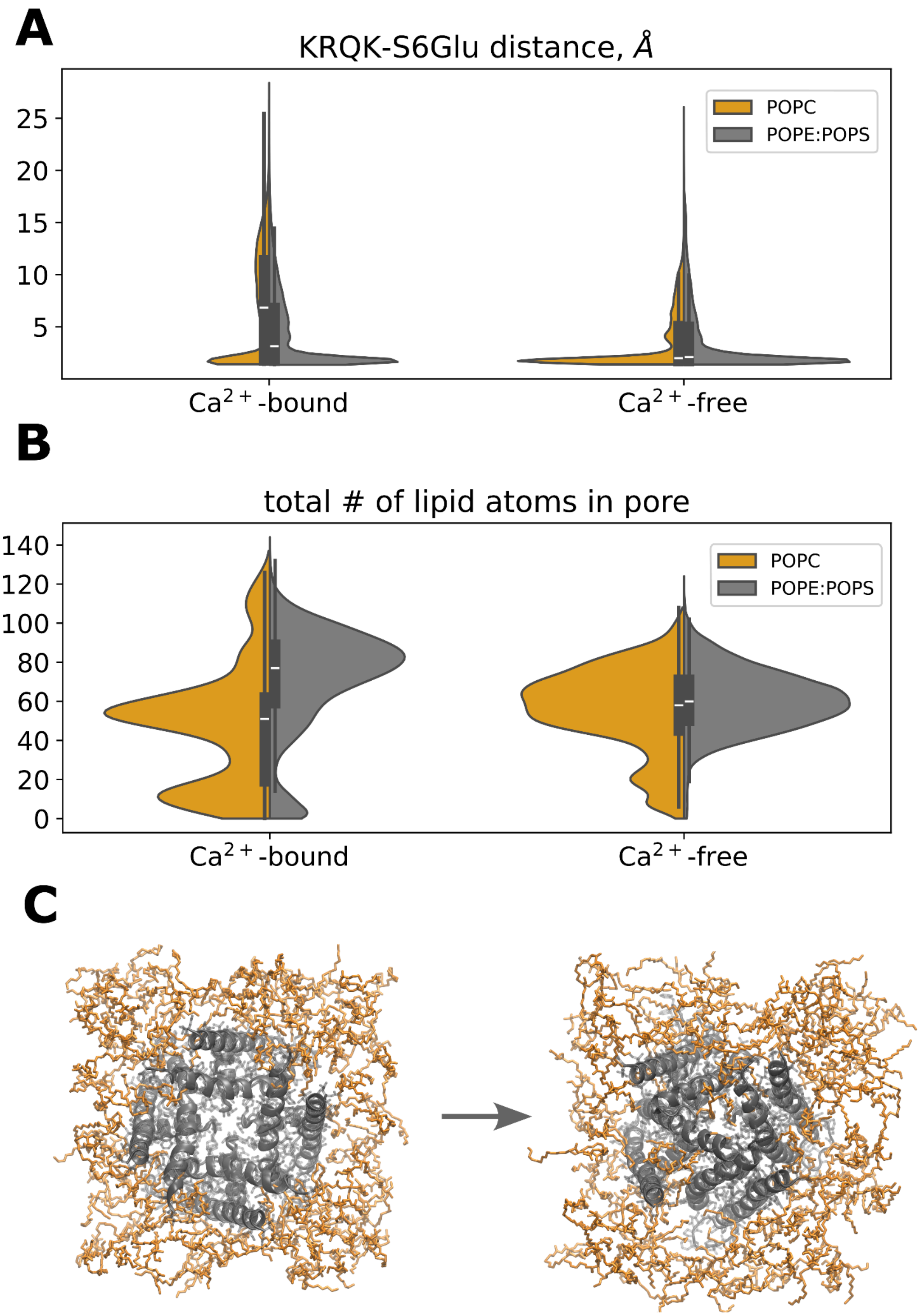

SI Fig. 11. Atomistic simulations of the isolated pore domain. Distributions of (*A*) distances between ^{317}KRQK^{320} and E310/E313, and (*B*) number of lipid atoms (non-hydrogen, out of ~50 per lipid molecule) in the pore. (*C*) Simulation snapshots showing low structural stability of the isolated pore domain and high propensity to be blocked by lipids regardless of the state of the channel (here, Ca^{2+}-bound is shown).

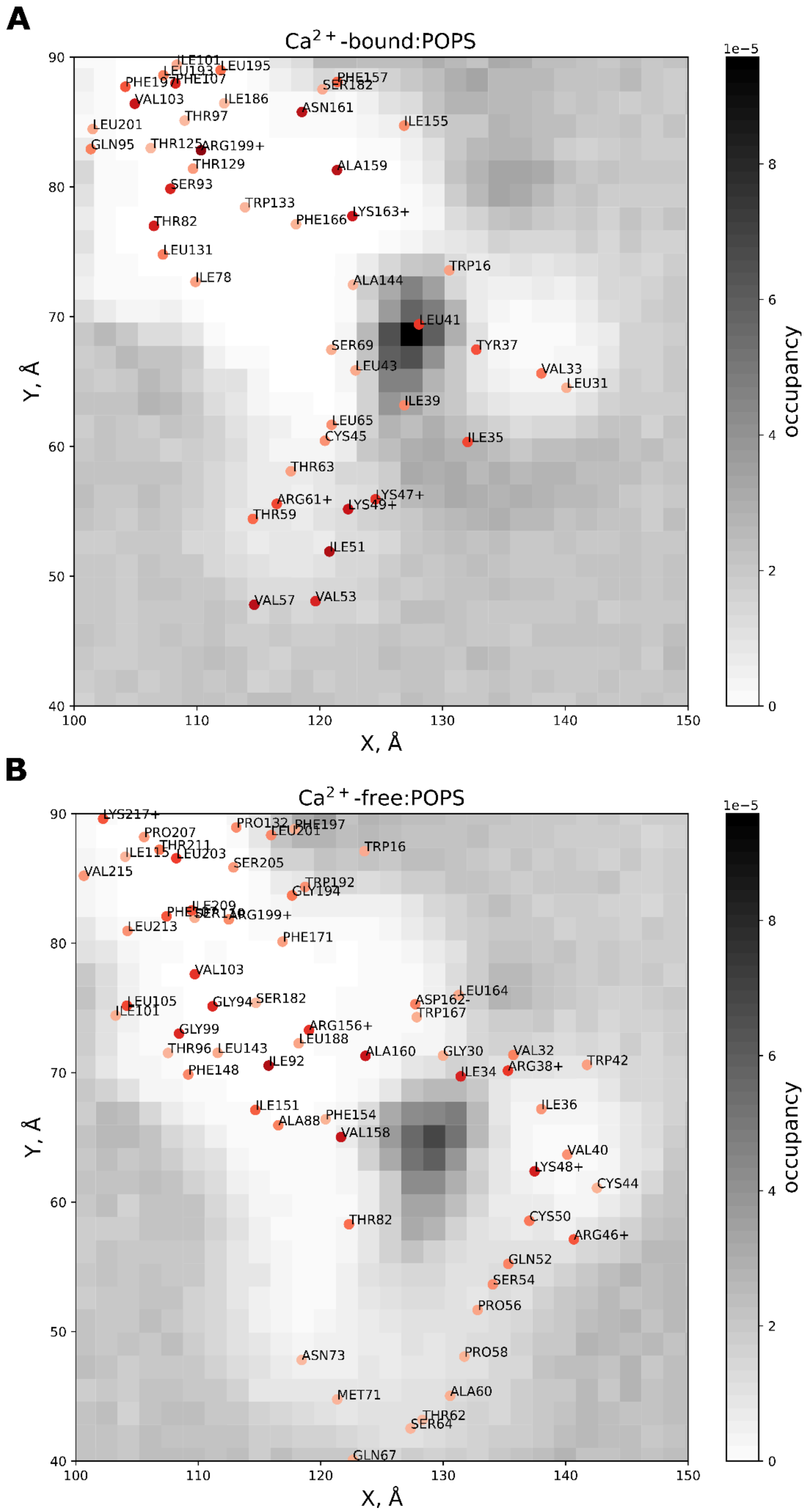

SI Fig. 12. Occupancies of PO4 beads of POPS near the binding site located in the voltage sensor in (*A*) Ca^{2+}-bound and (*B*) Ca^{2+}-free states, with protein residues interacting with POPS highlighted; darker color indicates more frequent contacts.

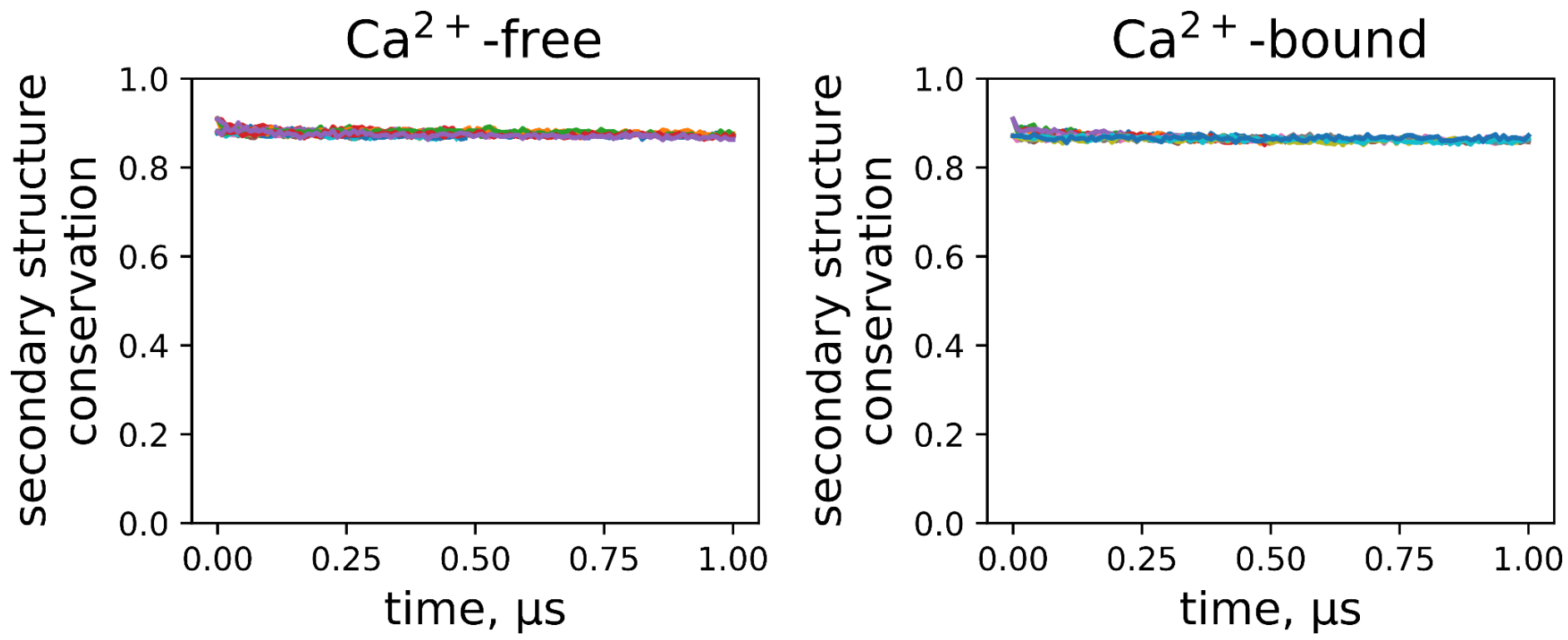

SI Fig. 13. Fraction of the residues with conserved secondary structure relative to Ca^{2+}-bound and Ca^{2+}-free CryoEM structures, respectively, as a general measure of BK conformational stability under simulation conditions. Different line colors correspond to different simulation conditions; all simulations performed are shown.

SI Table 1. *List of simulated systems discussed in the 'Results' subsections 'All-atom simulations of Ca^{2+}-bound and Ca^{2+}-free states' and 'Spontaneous closure of the open state'.*

**Channel structure**	**Membrane composition**	**Force field**	**Voltage**	**Duration**
Ca^{2+}-bound	POPC	CHARMM36m	0 mV	5x0.2..0.5 μs
			+300 mV	6x1 μs (started from of one of the 0 mV replicas)
Ca^{2+}-free	POPC	CHARMM36m	0 mV	5x0.2..0.5 μs
			+300 mV	3x0.2 μs (started from a 0 mV replica with no lipid block), 6x0.6..0.7 μs (started from two 0 mV replicas with partial lipid block, with 3 applied voltage replicas for each initial frame), 4x1 μs (started from a 0 mV replica with full lipid block)

SI Table 2. *List of simulated systems discussed in the 'Results' subsection 'Effect of membrane composition on lipid block'. Unless noted, listed simulations correspond to those of a full-length channel.*

Ca^{2+}-bound	POPE:POPS, 3:1	Martini3	0 mV	40 µs
Ca^{2+}-free	POPE:POPS, 3:1	Martini3	0 mV	40 µs (no restraints on water), ~20 µs (with restraints on water molecules preventing them from entering the pore)
Ca^{2+}-bound	POPC	Martini3	0 mV	40 µs
Ca^{2+}-free	POPC	Martini3	0 mV	40 µs (no restraints on water), ~20 µs (with restraints on water molecules preventing them from entering the pore)
Ca^{2+}-bound	POPE:POPS, 3:1	CHARMM36m	0 mV	4x0.2..0.5 µs (initial membrane configuration 1), 4x0.3..0.4 µs (initial membrane configuration 2)
Ca^{2+}-free	POPE:POPS, 3:1	CHARMM36m	0 mV	4x0.2..0.5 µs (initial membrane configuration 1), 4x0.2..0.3 µs (initial membrane configuration 2)
Ca^{2+}-bound, isolated pore domain	POPC	CHARMM36m	0 mV	10x1 µs

Ca^{2+}-free, isolated pore domain	POPC	CHARMM36m	0 mV	10x1 μs
Ca^{2+}-bound, isolated pore domain	POPE:POPS, 3:1	CHARMM36m	0 mV	4 different initial membrane configurations, 5x1 μs each
Ca^{2+}-free, isolated pore domain	POPE:POPS, 3:1	CHARMM36m	0 mV	4 different initial membrane configurations, 5x1 μs each
